# Unveiling the Dynamics of KRAS4b on Lipid Model Membranes

**DOI:** 10.1007/s00232-021-00176-z

**Published:** 2021-04-07

**Authors:** Cesar A. López, Animesh Agarwal, Que N. Van, Andrew G. Stephen, S. Gnanakaran

**Affiliations:** 1 Theoretical Biology and Biophysics Group, Los Alamos National Laboratory, Los Alamos, NM 87545 USA; 2 National Cancer Institute RAS Initiative, Cancer Research Technology Program, Frederick National Laboratory for Cancer Research, Leidos Biomedical Research, Inc., Frederick, MD 21702 USA

**Keywords:** Cancer, KRAS4b, Molecular dynamics

## Abstract

Small GTPase proteins are ubiquitous and responsible for regulating several processes related to cell growth and differentiation. Mutations that stabilize their active state can lead to uncontrolled cell proliferation and cancer. Although these proteins are well characterized at the cellular scale, the molecular mechanisms governing their functions are still poorly understood. In addition, there is limited information about the regulatory function of the cell membrane which supports their activity. Thus, we have studied the dynamics and conformations of the farnesylated KRAS4b in various membrane model systems, ranging from binary fluid mixtures to heterogeneous raft mimics. Our approach combines long time-scale coarse-grained (CG) simulations and Markov state models to dissect the membrane-supported dynamics of KRAS4b. Our simulations reveal that protein dynamics is mainly modulated by the presence of anionic lipids and to some extent by the nucleotide state (activation) of the protein. In addition, our results suggest that both the farnesyl and the polybasic hypervariable region (HVR) are responsible for its preferential partitioning within the liquid-disordered (Ld) domains in membranes, potentially enhancing the formation of membrane-driven signaling platforms.

**Supplementary Information:**

The online version contains supplementary material available at 10.1007/s00232-021-00176-z.

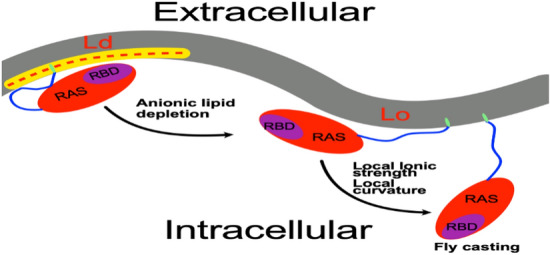

## Introduction

KRAS4b (Kirsten rat sarcoma viral oncogene homolog 4b) proteins are membrane-associated GTPases, responsible for regulating signaling pathways involved in cell growth and division (Hancock 2003; Hobbs et al. 2016; Ntai et al. 2018; Nussinov et al. 2018). Research has provided data that draws a mechanistic picture of KRAS4b function at the molecular level, including insights into its nucleotide exchange (Cromm et al. 2015; Prakash and Gorfe 2014) coupled with conformational cycling between “active” and “inactive” states (Carvalho et al. 2015). Such information has been relevant in understanding the phenotypic effects of oncogenic RAS gene mutations that lock the protein in the GTP-bound active state.

**Fig. 1:**
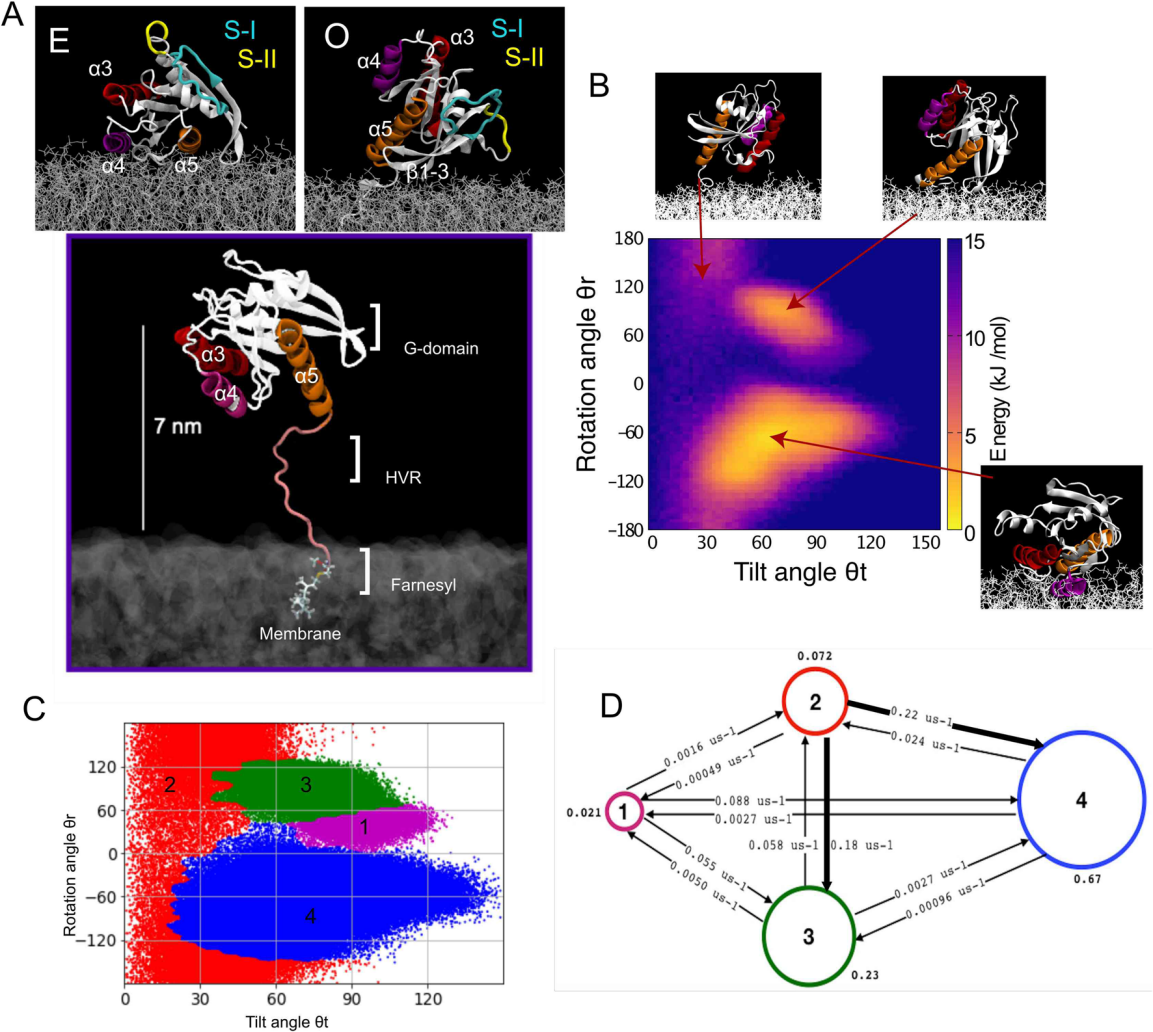
Dynamics of KRAS4b-GTP on anionic lipid membranes. **a** Schematic representations of the exposed (top left panel) and occluded (top right panel) conformations of KRAS4b bound to membrane. Switches I and II are highlighted in cyan and yellow colors, respectively. Representative initial configuration of KRAS4b (bottom panel) on an anionic membrane model (see methods). The G-domain is attached to the membrane via the farnesyl group. Initial conformation bias is alleviated by initiating the simulation with the G-domain placed 7 nm away from the membrane surface. Helices 5, 4 and 3 are colored in orange, purple, and red, respectively. The HVR is colored in pink. The conformation corresponds to a back mapped CG representation to AA to properly depict the secondary structure of the protein. **b** Tilt *θ*t and rotation *θ*r angle projection of KRAS4b on anionic lipid model. Insets show a representative conformation from the major basins. Helices 5, 4 and 3 are colored as in (**a**). The map was generated using a Boltzmann multi-dimensional histogram with 70–70 bin size as implemented in GROMACS. **c** The HMM analysis of coarse-grained MD trajectories was carried out using a framework built upon the architecture of MSM (see Methods). Using the same coordinates as in (**b**), four metastable states are obtained from MSM-HMM analyses. Here, the microstates are colored according to the macrostates they belong to. **d** The rates between macrostates from HMM analysis. Thicker arrows correspond to faster rates

Less is known about the conformational membrane-dependent orientations of KRAS4b and how its dynamics modulate the interaction with secondary effectors such as those involved in the Mitogen-activated protein kinase signaling (MAPK) pathway. In this respect, spectroscopy data have provided relevant insights into membrane-associated KRAS4b conformations (Erwin et al. 2017; Kapoor et al. 2012; Weise et al. 2011; Werkmuller et al. 2013). Pioneering work has shown that KRAS4b is associated in two main orientations on the membrane (Kapoor et al. 2012), modulated by its activation state (GDP vs. GTP), emphasizing the role of the G-domain in protein-membrane interactions. Paramagnetic relaxation enhancement (PRE) NMR have provided more insights on the conformations of membrane-associated KRAS4b (Fang et al. 2018; Mazhab-Jafari et al. 2015). Thus, experimental data confirmed the presence of two main configurations; one parallel to the membrane in which helices $\alpha$3, $\alpha$4 and $\alpha$5 enhance the association, and one orthogonal in which beta strands $\beta$1, $\beta$2 and $\beta$3 directly contact the membrane. The latter occludes switch I of KRAS4b, potentially hiding the effector binding site (Fig. 1a right and left top panels).

Computationally derived data have also been valuable in describing the conformation and dynamics of KRAS4b, confirming experimental findings (Chakrabarti et al. 2016; Li et al. 2018b; Mazhab-Jafari et al. 2015; McCarthy et al. 2016; Prakash and Gorfe 2013, 2014; Prakash et al. 2016; Travers et al. 2018). More specifically, molecular dynamic (MD) simulations suggest that the association of the G-domain to the membrane is lipid-dependent (Cao et al. 2019; Gregory et al. 2017; Prakash et al. 2016), highlighting the role of the C-terminal polybasic hypervariable region (HVR, residues 167–185) (Banerjee et al. 2016; Janosi and Gorfe 2010; Neale and Garcia 2018; Zhou et al. 2017) and its farnesyl group, both of which are involved in the recruitment of KRAS4b within the anionic-rich lipid domains of membranes (Brunsveld et al. 2009; Erwin et al. 2016; Jang et al. 2015; Plowman et al. 2008; Rowat et al. 2004; Vogel et al. 2009). A recent massive MD simulation (Neale and Garcia 2020) confirmed the lipid-modulatory effect on the dynamics of KRAS4b. Most importantly, the presence of anionic lipids affects the accessibility of the protein surface for the binding of RAS effectors (Neale and Garcia 2020). Similarly, it has been suggested that transitions between “available” and non-available” states occur within the microsecond-nanosecond time-scale (Prakash and Gorfe 2019), which is beyond the time scale of high-resolution experimental measurements.

Here we model, describe and quantify the membrane-bound orientation transitions of KRAS4b in lipid model membranes by coupling coarse-grained (CG) simulations with Markov State Models (MSM) and Hidden Markov Models (HMM). CG simulations provide biologically relevant time scales (milliseconds), which can be directly combined with MSM-HMM to estimate both metastable states and transitions between the major membrane-bound conformations. Our results confirm that KRAS4b membrane-dependent states and transitions between them are strongly influenced by lipid composition, the nucleotide loading state and the overall ionic concentration of the medium. When interacting with segregated lipid membrane phases, KRAS4b shows a preferential partitioning towards the anionic lipid-enriched phases, which is driven by both the farnesyl substituent and the HVR region of the protein.

## Simulation Details

### System Set-Up

#### Coarse-Grained (CG) Model of KRAS4b

The initial coordinates for KRAS4b was taken from the protein data bank (PDB 4G0N). These coordinates were transformed into CG beads using standard parameters based on the MARTINI 2.2 force field (Marrink et al. 2007). Two CG structures were generated to resemble the internal dynamics of either a GDP or a GTP state. For this reason, a different set of backbone elastic bands were applied to modulate the intermolecular dynamics of both switch I and II, as has been previously characterized (Spoerner et al. 2001). We validated our model once the overall dynamics of the protein was in good agreement with atomistic simulations generated locally.

We used specially derived parameters for the farnesyl group (Travers et al. 2018) and the dynamics of the CG HVR (residues 166–185) model was improved by iteratively modifying the angles connecting the backbone beads, based on long atomistic simulations. The HVR was considered fully disorder from residues 172–185, as observed in several crystal structures.

#### Coarse-Grained Model of Lipid Membranes

Several lipid environments were constructed using the ./*insane* script as part of the Martini lipidomic library (Wassenaar et al. 2015), provided by the Martini force field website (http://cgmartini.nl). First, a CG membrane containing 70:30 1-palmitoyl-2-oleoyl-glycero-3-phosphocholine:1-palmitoyl-2-oleoyl-glycero-3-phospho-L-serine (POPC:POPS) (224 lipids per leaflet) was placed in a triclinic box and fully solvated with standard Martini water beads. The normal vector of the box was extended up to 12 nm from the membrane surface to allow full dynamics of the protein. Once the farnesyl of the KRAS4b CG model was embedded in the membrane, the system was neutralized and an overall concentration of 150 mM Na^+^/Cl^−^ ions was added. Although K^+^ is the most abundant intracellular cation, there is no distinction in its representation at the Martini resolution. A larger version of this system (1300 lipids per leaflet) was also constructed and care was taken to maintain the same overall ionic strength. The same protocol was used for constructing a neutral 100% POPC system and a small liquid-ordered (Ld) membrane containing 60% 1,2-dipalmitoyl-sn-glycero-3-phosphocholine (DPPC) and 40% cholesterol.

A coexisting liquid-ordered/liquid-disordered (Lo/Ld) membrane was also built by randomly placing 40% DPPC, 25% DOPC, 30% cholesterol and 5% DOPS within the XY plane of a triclinic box. The same lipid mixture has been computationally validated as a good representative of a phase-segregated system (Sodt et al. 2015). The final model of the membrane was composed of 2014 lipids per leaflet and a total of 57,768 Martini water beads (230 K atomistic waters). Like the smaller systems, the farnesyl group of the KRAS4b CG model was embedded in the membrane and the total charges of the system was neutralized and an overall 150 mM Na^+^/Cl^−^ ions was added. 0.1% of water beads were replaced with antifreeze particles to avoid the artificial freezing of the solvent. Lipids were represented using an optimized set of parameters as previously published (Carpenter et al. 2018; Travers et al. 2018).

### MD Protocol

#### CG MD Simulations

The GROMACS MD engine (version 5.1.4) (Páll et al. 2015) was used for integrating the equations of motion during the CG simulations. We followed a recent update in CG parameters set-up for performing the simulations (de Jong et al. 2016), which proved to be overall more efficient in preserving the internal energy. The simulations used a 30 fs time-step. Reaction-field electrostatics were used with a Coulomb cut-off of 1.1 nm and dielectric constants of 15 or 0 within or beyond this cut-off, respectively. A cut-off of 1.1 nm was also used for calculating Lennard Jones interactions, using a scheme that shifts the Van der Waals potential to zero at this cut-off. The smaller lipid bilayers were coupled to a constant thermal bath, which was maintained at 310 K via separate coupling of the solvent and membrane/protein components using a velocity rescaling (Bussi et al. 2007) thermostat with a relaxation time of 1.0 ps. However, the coexisting lipid model (raft membrane) was coupled to 290 K to promote lipid phase separation (Sodt et al. 2015). Semi-isotropic pressure coupling was set for each system at 1 bar using a Berendsen (Berendsen et al. 1984) barostat during a short equilibration step and later exchanged to a Parrinello-Rhaman (1980) approach with a relaxation time of 12.0 ps. Simulations of two-lipid mixtures were run for a total of 1 ms (10 replicates of 1 $\mu$s each). Simulations of lipid segregated mixtures were run for a total of 0.5 ms (10 replicated of 0.5 $\mu$s each). Trajectories were saved every 3 ns for subsequent analysis.

### Free Energy Calculations

#### Metadynamics

In order to enhance the convergence of the membrane-farnesyl interaction free energies, we set a metadynamics (Barducci et al. 2011) protocol making use of both parallel-tempered and the well-tempered approach.

In a regular metadynamics calculation, a set of collective variables (CV) are affected by a bias potential so a full reaction coordinated energy landscape connecting a process can be described in terms of its change in free energy. The bias potential is built as a sum of Gaussian kernels deposited along the trajectory of the CVs. In a well-tempered approach (Dama et al. 2014), the Gaussian heights are affected during the simulation time according to:

$$W\left(k\tau \right)={W}_{0}\mathrm{exp}(-\frac{V\left(s\left(q\left(k\tau \right)\right),k\tau \right)}{{k}_{B}\Delta T})$$,

where W_0_ is an initial Gaussian height and ΔT corresponds to the dimension of the temperature. In order to compensate for the underlying free energy, it is necessary to apply a Bias term in the form of:

$$\gamma =\frac{T+\Delta T}{T}$$,

which is the ratio between the temperature of the CVs (T+$\Delta T)$ and the temperature of the system. Often, change in free energies is hard to converge due to the large hysteresis of the system, or due to a complicated energetic path. To overcome this situation, the well-tempered metadynamics can be effectively combined with the replica exchange protocol. Thus, a single metadynamics calculation can be performed by *N* replicated in parallel and at different temperatures, using the same set of CVs. The parallel-tempered (PT) acceptance probability was modified to account for the presence of the bias potential:

$${\Delta }_{ij}^{PTMetaD}={\Delta }_{ij}^{PT}+\frac{1}{{k}_{B}{T}_{i}}\left[{V}_{G}^{i}\left(s\left({R}_{i}\right),t\right)-{V}_{G}^{i}\left(s\left({R}_{j}\right),t\right)\right]+\frac{1}{{k}_{B}{T}_{j}}\left[{V}_{G}^{j}\left(s\left({R}_{j}\right),t\right)-{V}_{G}^{j}\left(s\left({R}_{i}\right),t\right)\right]$$

where ${V}_{G}^{i}$ and ${V}_{G}^{j}$ are bias potentials of the *i*th and *j*th replicas, respectively.

In our free energy calculations, we have biased two specific reaction coordinates (CVs), namely the center of mass (COM) of the farnesyl with respect to the COM of the membrane and an angle defining the relative orientation of the molecule with respect to the membrane. The later was defined as the angle from between bead-1, bead-4 and the COM of the membrane. In either case, a Sigma value of 0.1 nm and 0.35 radians was applied to the COM-COM distance and the angle, respectively. We found a bias factor of 15 was enough to converge the simulations. We found a combination of a CG model with the PtWtMET allows good convergence within 0.5 us of MARTINI time scales.

### Umbrella Potential

In order to compute the required free energy for desorbing the HVR region from a small membrane patch, we used the umbrella sampling approach (Kästner 2011) on the CG systems. The HVR consists of residues 166 to 185 of the KRAS4b sequence, in which the farnesyl group was removed. Both the N and C termini were represented in their charged state. A total of 100 independent windows per system were used, which were spaced 1 Å apart. A restraining potential of 1000 kJ mol^−1^ nm^−2^ was applied to the center of mass of the HVR region with respect to the center of mass of the membrane and along the normal (*z*) coordinate. For each window, 5 μs long simulations were performed. The desorption free energy was reconstructed using the weighted histogram approach (Hub et al. 2010) as implemented in GROMACS and convergence was assessed via block averaging by dividing the trajectory in three independent blocks (1 μs each).

### Markov State Models (MSM) and Hidden Markov Models (HMM)

To describe the orientational states of KRAS4b with respect to membrane, we used Hidden Markov Models (HMMs) (Noe et al. 2013), which build upon the architecture of Markov state models (MSMs) (Pande et al. 2010; Prinz et al. 2011; Schütte et al. 1999). By choosing a pre-defined reaction coordinates to characterize the orientations of KRAS4b with respect to the membrane, MSMs estimate the optimal number of metastable states that can describe the dynamics of the system. In MSM’s, the state space is discretized into *n* microstates and the system’s dynamics is modeled by a *nxn* transition probability matrix where an element in the transition matrix represents the probability of jumping to state *j* at time *t (*also called lag-time*)* from state *i.* The implied timescales can be obtained from the eigen values of this transition matrix as a function of lag-time. If there is a separation of timescale between *M*th and (*M* + 1)th timescale, then the dynamics can be described by ‘*M*’ metastable states. In this work, the state space is discretized into 400 microstates using k-means clustering algorithm (Hartigan and Wong 1979). MSM analysis was initiated by computing the relaxation timescales as a function of the time resolution of the model. For most cases considered in this work, the timescales reached a plateau at around 450 ns. Based on this analysis, we then employ Perron Cluster–Cluster analysis (PCCA) (Deuflhard and Weber 2005) to obtain four metastable states in the system.

In the MSM, the rates between the identified states hinges on the input subspace and the quality of discretization. Thus, results obtained from MSMs may differ with different order parameters and clustering methods. We overcame this limitation using HMMs on a *M* × *M* transition matrix describing the dynamics between the metastable states identified using MSM. Then, the resulting probability matrix with dimensions *M* × *n*, where the row vector gives the probability that the metastable state will correspond to one of the *n* discrete states. Generally, it is estimated by the Baum-Welsch Expectation–Maximization algorithm (Baum et al. 1970). Even though all thermodynamic and kinetic properties calculated from HMM can also be computed using MSM, it has been shown that the metastable dynamics can be exactly described using HMMs even with a poor discretization quality. All MSM/HMM construction and analysis in this work were performed with the PyEmma software package (Scherer et al. 2015).

## Results

### KRAS4b, Dynamics and Configurations

Our initial studies focused on the effect of lipid membrane composition on the dynamics of KRAS4b. In order to enhance the sampling and allow the exploration of otherwise prohibited time scales (milliseconds) at atomic level, we used the reductive, yet efficient Martini CG model (Marrink et al. 2007). Our first system consists of a pre-equilibrated 70:30 POPC-POPS lipid membrane patch and a model of GTP-bound KRAS4b protein (see Methods) attached to the membrane via its farnesyl group. Initially, the G-domain protrudes 7 nm out of the water-membrane interface, while the HVR serves as a linker between the farnesyl group and the globular G-domain (Fig. 1a). Figure 1b summarizes our findings of KRAS4b conformations in this membrane mixture, in terms of protein orientation based on two previously well-characterized angles (Neale and Garcia 2020; Travers et al. 2018): the tilt angle of the G-domain away from the bilayer normal, *θ*t, and the azimuthal angle at which that tilt occurs (i.e., the rotation), *θ*r. Direct projection of both angles (Fig. 1b) recapitulates the presence of two major basins of strong density: the most extended and stabilized by ~  − 15 kJ mol^−1^ is featured by *θ*t 30°–120° and *θ*r (− 20°) to (− 160°), and a second narrower basin localizes between *θ*t 45°–90° and a *θ*r 30°–120°. Projection of both *θ*t and *θ*r also allows the identification of a third state, although it represents a minor population compared with the other two basins. Representative protein orientations from each basin are shown as small insets (Fig. 1b). The biggest basin is characterized by close contacts of helices 5 (orange helix), 4 (purple helix) and 3 (red helix) of the G-domain with the membrane. The large variation in tilt angle for this basin indicate helices 3 and 5 are able to weakly interact with the membrane. This conformation is also featured by the full exposure of the effector binding site to the bulk water and was previously identified as the ‘exposed’ state (Mazhab-Jafari et al. 2015). The second basin is highlighted by the direct interaction of beta strands 2 and 3 with anionic lipids and the occlusion of the effector binding site (‘occluded’ state).

To further characterize the metastable states, we performed an MSM analysis. In this work, the state space represented by both *θ*t and *θ*r is discretized into 400 microstates using the k-means clustering algorithm (Hartigan and Wong 1979). MSM analysis is then initiated by computing the relaxation timescales and a plateau is reached at around 450 ns, indicating that a robust estimation of the relaxation processes beyond the first three dominant processes cannot be carried out. Thus, we construct an MSM transition matrix using a lag-time of 450 ns and three dominant eigenvectors were obtained from the transition matrix analysis (Fig. S1A). Based on this analysis, we employ a Perron Cluster–Cluster analysis (PCCA) (Deuflhard and Weber 2005) to obtain four metastable states in the system. These four basins represent the orientations of KRAS4b with respect to the membrane (Fig. 1c): first, the largest populated states (state 4 and 3), covering 67% and 23% of the entire simulation trajectory, respectively; and a second boundary constituted by low populated regions (states 1 and 2), covering ~ 10% of the total accessible states.

Then, by applying HMM analysis (see methods) on the *M* × *M* transition matrix, we quantify the transitions among these four states (Fig. 1d). This analysis not only allows us to identify the most probable pathways between the different states, but the time scales involved in such transitions. Accordingly, the transition from exposed (state 4) towards occluded (state 3) will first require access to the red region (state 2), characterized by a partial membrane detachment and unspecific tilt and rotation angles of the G-domain. In this scenario, avoiding state 2 clearly hampers the exposed-occluded transition by a kinetic barrier of nearly two orders of magnitude (3 → 4: 0.0027 $\mu$s^−1^ and 4 → 3: 0.00096 $\mu$s^−1^), becoming thus inefficient (e.g. direct jump) (Fig. 1d**)**.

An NMR-MD integrative study has been published (Van et al. 2020) which analyzed the dynamics of KRAS4b on anionic membranes. Computational derived Paramagnetic resonance enhancement (PRE) profiles resembled those obtained experimentally. Thus, this comparison showed the predictive property of the CG modeling and provided confidence for the conclusions obtained from our simulations. We have also compared our results with the orientations of previously deposited models (Fang et al. 2018; Mazhab-Jafari et al. 2015) in the protein data bank. These models were obtained using molecular docking (HADDOCK) in combination with distance restraints obtained from PRE-NMR data. We compared these models to our simulations by projecting their tilt and rotation angles onto our conformation map in Fig. S1B. The authors classified these conformations as Exposed3 (blue circles), Exposed (red circles), and the Occluded (cyan circles) (Fang et al. 2018; Mazhab-Jafari et al. 2015). From the comparison, the conformations are indeed sampled by our simulations, especially the Occluded and Exposed3. The major basin (state 4) sampled by our CG simulations is broad enough to contain both exposed states, but it is more complex in terms of dynamics. In fact, MCM analysis reveals that the total extent of state 1 cannot be recapitulated by the published conformations (Fig. S1C).

### KRAS4b Dynamics and States are Impacted by Lipid Composition

**Fig. 2:**
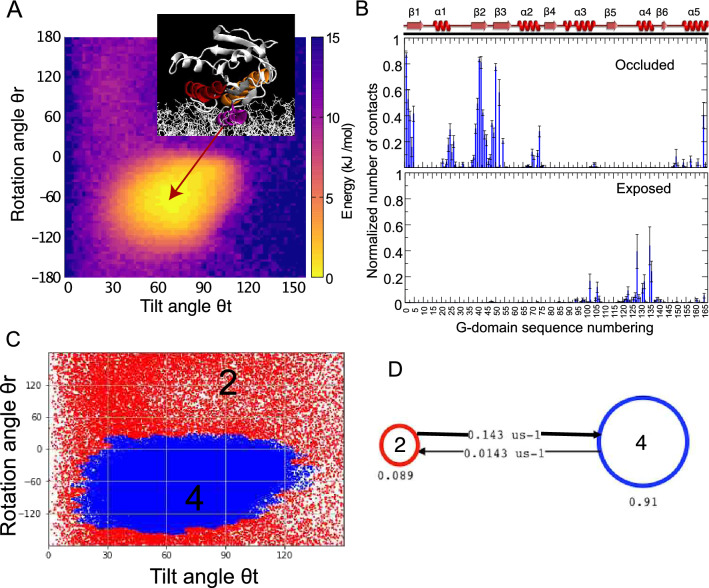
Dynamics of KRAS4b-GTP on a zwitterionic membrane. **a** Projection of Tilt *θ*t and rotation *θ*r angle show the presence of the “exposed” state in pure POPC bilayer. Inset highlights a representative structure from the strongest density region in the map. **b** Normalized number of contacts between KRAS4b and the membrane. Analysis was carried in anionic membranes containing 30% POPS using as a reference any sidechain bead of the protein within 0.55 nm of any bead of the anionic lipid. Contacts were normalized by the total simulation time. **c** Using the same coordinates as in (**a**), two metastable states are obtained from MSM/HMM analyses. The microstates are colored according to the macrostates they belong to. **d** The rates between macrostates from HMM analysis. Thicker arrow corresponds to the fastest transition rate of the system

Membrane composition has a big impact on the dynamics of KRAS4b. This is verified by simulations of GTP-bound KRAS4b in pure POPC bilayers shown in Fig. 2a. The absence of anionic lipids eliminates the preference for the occluded conformation, and lead to the stabilization of only the “exposed” configuration. In fact, our analysis suggests that in the “occluded” state, the G-domain is stabilized by several contacts with anionic lipids. These contacts are persistently observed for residues 1–5 and 36–50, accounting for almost 50% of the time when KRAS4b is in a POPC:POPS mixture (Fig. 2b).

As expected, a similar analysis using MSM-HMM (Fig. S2A) on the coarse-grained dynamics of KRAS4b in non-anionic membranes reveals only two states, with no presence of the occluded state (Fig. 2c, d). Indeed, the exposed state covers 91% of the entire simulation time, with suddenly scattered transitions towards non-specific conformations (Fig. 2c). The slow transition (0.0143 μs^−1^) describes the transition towards state 1 (non-specific), however, this is a short-lived state and the protein rapidly moves back to the “exposed” configuration (Fig. 2d). This result clearly suggests that anionic lipids play a key role in maintaining the “occluded” state, supporting the notion of a ‘membrane-modulatory’ activity on KRAS4b.

The G-domain of KRAS4b makes specific contacts with anionic lipids in the occluded state (Fig. 2b), however, it is not clear how the exposed state is stabilized in zwitterionic membranes. Thus, we ask the question whether RAS dynamics can also be affected by the presence of the HVR. Several residues in the HVR maintain a persistent interaction with anionic lipids, especially the polybasic stretch. Although the membrane-contact profile of HVR seems to be state-independent, clear differences associated with residues 167, 168–169, and 171–177 were observed (Fig. S2B). To determine whether this relative difference is associated with the occluded and exposed states, we computed the state-dependent intramolecular interactions between all residues of KRAS4b. This analysis reveals specific contacts between the HVR and the G-domain (Fig. S2C). Three hot spots are detected as potential regions of interaction between the G-domain and the HVR. A very consistent interaction between loop7 (residues 102–108) and the polybasic residues of the HVR is present, regardless of the nucleotide-bound state (Fig. S2D). Unexpectedly, additional simulations, in which residues 102–110 of loop7 are mutated to Alanine residues confirmed that this interaction destabilizes the occlusion of the G-domain (Fig. S2E). In fact, interactions between residues 135–140 of the G-domain with the HVR are key to provide extra anchoring for the stabilization of the G-domain in the exposed state (Fig. S2C enclosed blue circle).

Next, we analyze the impact of lipid composition on the lateral dynamics of KRAS4b. More precisely, we have computed the lateral mean square displacement of the protein, for the two primary states, exposed and occluded. As shown in Fig. S3A, there is a negligible difference between the two states in terms of lateral diffusion, correlated with the relative homogeneity of the membrane. No differences in the lateral diffusion of the lipids were observed either.

### KRAS4b Activation State Affects Its Dynamics

**Fig. 3:**
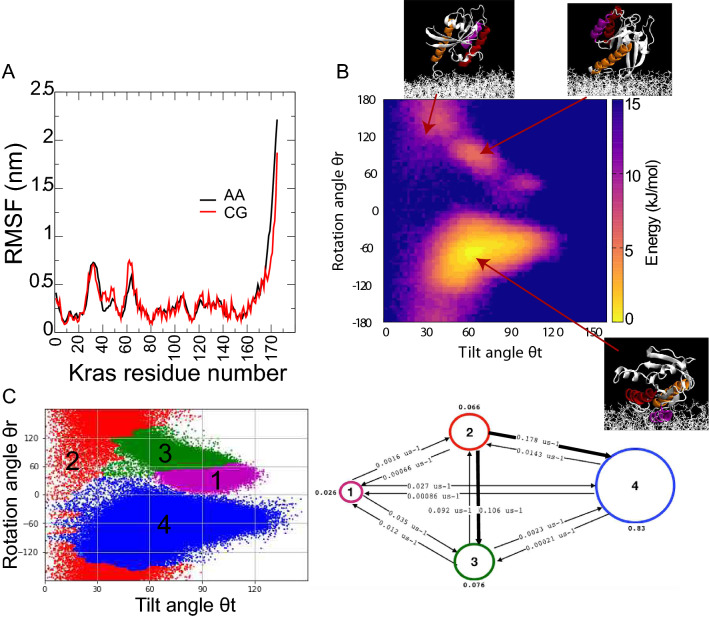
Effect of nucleotide on KRAS4b dynamics. **a** Internal backbone fluctuation between an atomistic model and a parametrized GDP-loaded CG model (see methods). There is good agreement in fluctuations within switch I and II. **b** Tilt *θ*t and rotation *θ*r angle projection of GDP-bound KRAS4b on anionic-rich membranes. **c** Using the same coordinates as in (**b**), four metastable states are obtained from MSM-HMM analyses. The microstates are colored according to the macrostates they belong to. **d** The rates between macrostates from HMM analysis. Thicker arrows correspond to the fastest rates of the system

Previous studies suggest that KRAS4b conformation on membranes is affected by the nucleotide state (Kapoor et al. 2012; Mazhab-Jafari et al. 2015). In fact, PRE experiments confirm that the interaction of KRAS4b with the membrane is modulated differently by the GDP- and GTP-bound nucleotide (Mazhab-Jafari et al. 2015). The authors suggested that GDP partially destabilizes the conformations of switches I and II, thus affecting the interaction of these regions with the membrane. This hypothesis has been confirmed using long time-scale simulations (Pantsar et al. 2018; Shima et al. 2010; Spoerner et al. 2001), supporting the notion of a conformational change of KRAS4b, which is dependent on the nucleotide state (GTP vs GDP). However, one open question is whether this perturbation in the structure of RAS could directly influence its dynamic on a membrane. Thus, we have built a CG model of the GDP-bound KRAS4b by modulating the fluctuations within switch I and II as captured from AA simulations (Fig. 3a), and probed its dynamics in the presence of a 70:30 POPC:POPS anionic membrane model.

A summary of the conformations of GDP-bound KRAS4b is shown in Fig. 3b from a total of 1 ms simulation time (10 replicas 0.1 ms each). At first glance, direct projection of *θ*t and *θ*r does not show any major difference with respect to the GTP-bound protein in Fig. 1b. The energy landscapes of KRAS4b-GDP and KRAS4b-GTP look similar. However, further analysis using MSM-HMM (Fig. S3B, Fig. 3c, d) confirms differences in the relative populations for each state, as well as their transitions. First, the population in the “exposed” state (state 4) rises by almost 20% when compared with the GTP-loaded protein. This increase in the exposed configuration is compensated by a decrease of the occluded population, which drops to nearly ~ 1%. Although the GDP-loaded model can form specific contacts with PS lipids, they are weaker when compared to GTP-loaded KRAS4b.

Interestingly, transition rates are similar along the different conformation pathways when compared with GTP-loaded, however, there are important noticeable changes. For instance, the forward path transition from state 4 to 1 is clearly hampered by almost an order of magnitude, suggesting that transitions towards state 3 along this path are prohibited. Similarly, all transition rates around state 3 disfavors the stabilization of this basin. Thus, we can conclude that indeed GDP influences the dynamics of KRAS4b on anionic membranes, disfavoring both the thermodynamic and kinetic stabilization of the “occluded” state.

### Effect of Membrane Structure and Divalent Cations on KRAS4b Membrane Association

Clearly, anionic lipids have a key role in stabilizing the association of the G-domain on the membrane surface. However, their dynamics and interplay with the protein can be dramatically affected by both the size of the membrane and the content of divalent cations concentration. Therefore, we evaluated these effects on the stabilization of the G-domain with the membrane by running simulations of KRAS4b in two additional systems. First, a larger 70:30 POPC:POPS membrane system (see methods) was constructed in which the total ionic strength was maintained (150 mM Na^+^/Cl^−^). In addition, this large membrane patch could freely undulate, mimicking the behavior of a larger plasma membrane. Second, we replaced the 150 mM Na^+^/Cl^−^ of our original 70:30 POPC:POPS small membrane system by 75 mM Ca^++^ and 150 mM Cl^−^ (1–2 ratio) to test the effect of ionic strength change by the addition of divalent cations.

**Fig. 4:**
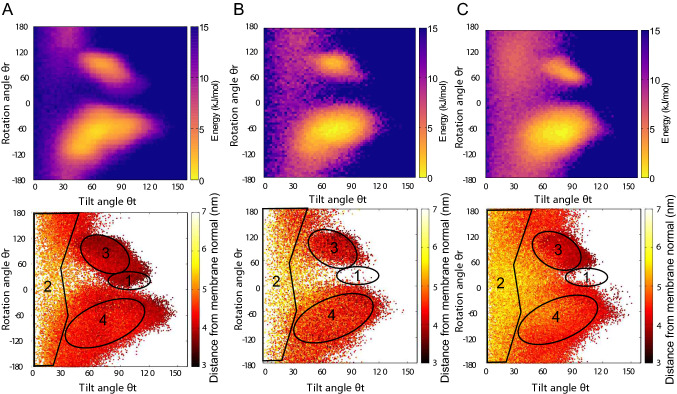
Effect of membrane structure and divalent cations on KRAS4b dynamics. **a** Tilt *θ*t and rotation *θ*r angle projection map for a regular 70:30 POPC:POPS box system (see methods). The corresponding orthogonal distance of the G-domain with respect to the membrane COM is shown in the bottom row. Numbers and areas correspond to states as calculated using MSM in Fig. 1. **b** Same as (**a**) but values were calculated from simulations of KRAS4b on larger membranes (see methods). Density shows a population increase of state 2. **c** Similar to (**a**) but ionic strength was provided by a concentration of 75 mM Ca^++^ and 150 mM Cl^−^ to test the effect of divalent cations. There is an increase in the “membrane-distal” state (state 2)

Our results indicate that system size, especially the undulations observed in the membrane, can indeed affect the behavior of KRAS4b (Fig. 4), even when anionic lipid ratio remains unchanged. This is more noticeable when calculating the orthogonal distance of the G-domain center of mass (COM) with respect to the membrane middle plane (see methods). In our regular small membrane system (Fig. 4a bottom inset) regions corresponding to both the exposed and occluded states (states 4 and 3, respectively) are closer to the membrane surface. On the other hand, in the large membrane, state 2 shows significantly more detachment from the membrane surface (Fig. 4b state 2), an important feature for allowing the transitions between the major basins. Although states 3 and 4 are still present, they are lower in intensity compared to the regular small system. Surprisingly, state 1 fully disappears suggesting that the stabilization of this state is directly impacted by the presence of membrane curvature.

Contrary to the effect with Na^+^ ions (Fig. 4a), divalent cations have stronger destabilizing effects as they directly compete for the association with anionic lipids (Fig. 4c) and can also directly disrupt interactions between the G-domain and the HVR. Consequently, both the exposed and occluded configurations are largely affected, although the later seems to be more impacted by divalent cations. The distance map shows that KRAS4b remains longer within the transition area, and more effectively desorbed from the membrane surface.

**Fig. 5:**
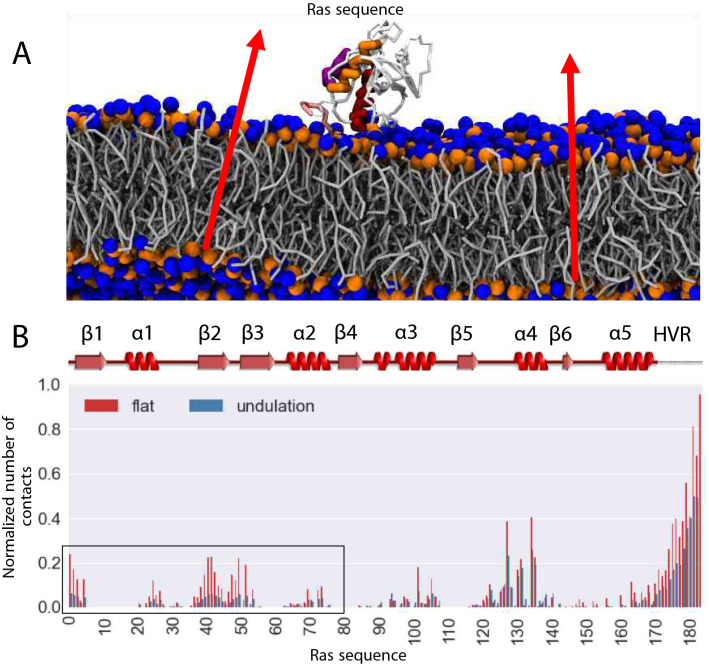
Membrane undulations destabilize KRAS4b adsorbed states. **a** Snapshot of KRAS4b on a large 70:30 POPC:POPS box system (see methods). KRAS4b is colored to highlight $\alpha$5 (orange), $\alpha$4 (purple), and $\alpha$3 (red) helices. Lipid tails are colored in gray while both the choline and phosphate groups are represented as blue and orange spheres, respectively. Red arrows indicate the direction of the membrane normal, highlighting the presence of local undulations. **b** Normalized number of contacts between KRAS4b and anionic lipids on the large membrane. Analysis was carried out for both the small system (flat) and large membrane (undulation) using 0.55 nm as an interaction distance cutoff. A representation of the protein secondary structure allows the identification of interactions stabilizing the occluded state which has affected the most (open black rectangle)

The mechanism for destabilization by divalent cations can be explained, however, it is not straightforward to provide an explanation for the disruptive mechanism observed in larger systems under the same Na^+^/Cl^−^ ionic strength. RAS-membrane proximity is affected by a decreased number of interactions with the surrounding lipids. Like the protein membrane-contact profile shown in Fig. 2b, we can explore the effects of curvature on RAS-membrane interactions in larger membrane patches. As expected, simulations of KRAS4b on larger membrane patches are featured by markedly larger undulations (Fig. 5a), otherwise absent in small patches. A contact analysis profile confirmed that protein-lipid contacts are affected, especially the interactions stabilizing the occluded ($\beta 1, \beta 2, \beta 3, \alpha 1 \text{ and } \alpha 2)$ conformation (Fig. 5b). It is important to note that the HVR-lipid contacts are mostly preserved, regardless of the membrane size (residues 170–185). Based on this result, we can conclude that undulations present in the larger membrane can disrupt most of the interactions responsible for stabilizing the occluded configurations (states 1 and 3), and subsequently promote RAS G-domain detachment from the water-membrane interface. In summary, our computational results indicate ionic strength as well as local deformations in the membrane can also modulate the conformations of KRAS4b, favoring the partial desorption of the G-domain.

### KRAS4b Localizes into Liquid-Disordered Membrane Domains

Our simulations suggest that the dynamics of KRAS4b is modulated by anionic lipids, the nucleotide-bound state and ionic concentration. It is unclear, however, whether its dynamics can be further modulated by the presence of plasma membrane-like lipids. In particular, we wanted to understand the molecular details governing its preferential localization in non-raft regions of the membrane (Weise et al. 2011). We first investigate the effect of cholesterol on the dynamics of KRAS4b. The projection of the tilt rotation angles for KRAS4b-GTP in a liquid-ordered (Lo) mimic is shown in Fig. S3C right panel. The density map shows that KRAS4b adopts similar conformations (exposed) as observed in the case of pure POPC lipids (Fig. 2a), corroborating the effects of anionic lipids. These results allow us to conclude that KRAS4b conformations are mainly impacted by the presence of anionic lipids, rather than membrane fluidity.

**Fig. 6:**
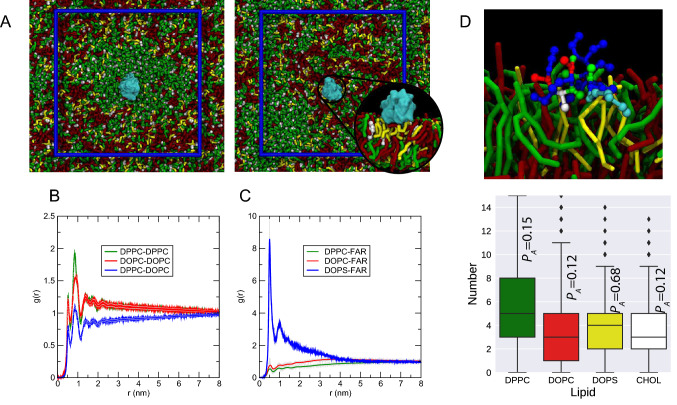
KRAS4b localizes in the anionic-rich domain of plasma membrane models. **a** Initial (left panel) and final (right panel) conformations of KRAS4b embedded on a de-mixed membrane system (see methods). 10 replicates were independently run for 50 us. DPPC is colored in green, DOPC in red, DOPS in yellow and CHOL in white. KRAS4b is colored in cyan. Note the presence of saturated lipid-enriched regions (green). The blue square represents the boundaries of the simulation box. **b** Lateral radial distribution function (g(r)), highlighting the preferential association of lipids. g(r) was averaged from the 10 independent simulations. The blue line highlights the lateral segregation between DPPC and DOPC. **c** g(r) highlighting the preferential lateral association of KRAS4b with anionic lipids. The farnesyl “FAR” group was taken as a reference for the calculation. **d** Close-up view of the association between the HVR region of KRAS4b and anionic lipids. Beads are colored according to amino acid type: blue-basic, red-acid, green-polar, white-hydrophobic, and cyan-nonpolar. Lipids are colored as in (**a**). Preferential lipid partitioning constant (see text) for the HVR in a mixed membrane (bottom panel)

Next, we focus on understanding the lateral partitioning of KRAS4b in a phase-separated membrane environment. Figure 6 summarizes the preferential localization of KRAS4b in a liquid-ordered/liquid-disordered (Lo-Ld) membrane patch composed of 40:25:5:30 DPPC:DOPC:DOPS:cholesterol. Initially, the protein is placed in a cholesterol-rich patch, where DOPS is poorly concentrated (Fig. 6a left panel). However, its dynamics in combination with the preferential association of the HVR for anionic lipids displaces KRAS4b close to the enriched anionic domain (Fig. 6a right panel). This behavior was consistently observed in ten replicates and was independent of the initial starting configuration of the protein. At the end of each simulation replicate (50 μs), the protein is found co-localized within the region where DOPC-DOPS lipids are enriched (e.g. Ld), and highlighted as a small inset in Fig. 6a. Similar to our previous simulations in two-lipid membranes, most of the DOPS interacting with KRAS4b are found strongly associated to the HVR segment. These interactions can also be captured by a set of radial distribution functions (RDFs), Fig. 6b. The respective RDFs between DPPC and DOPC suggest that these lipids are forming their coexisting regions (e.g. lipid-enriched domains) and are stable along the simulation time. On closer inspection, however, KRAS4b is preferentially surrounded by PS lipids as denoted by the higher peak within the first solvation shell (Fig. 6c**,** blue line) and to a much lower extent by DOPC and DPPC (in this order) lipids.

It has been reported that different RAS isoforms co-localize in different domains of a phase-separated membrane (Abankwa et al. 2007; Barcelo et al. 2013; Li et al. 2018a, 2012; Li and Gorfe 2012; Vogel et al. 2014). Thus, HRAS has been reported to co-localize within the Lo domains (Gorfe et al. 2007), whereas KRAS4b co-localized to Ld domains (Weise et al. 2011). It is interesting to note the small variation of the G-domain among RAS families, both structurally and in sequence; however, clear differences are observed at the level of both the HVR sequence and the post-translation modification at the C-termini. To investigate the role the G-domain and the farnesylated HVR play in partitioning between these lipid domains, we ran independent simulations of an anchored HVR (residues 166–185 of KRAS4b) in the same Lo-Ld membrane system (Fig. 6a). As expected, the HVR by itself preferentially co-localize within the Ld domains, which is enriched in anionic lipids (Fig. 6d). The preferential lipid partitioning (*P*_*A*_) of the HVR for the different components of the bilayer can be calculated with the following equation (de Jong et al. 2013):

$$P_{A} = \frac{{C_{A} /n_{A} }}{{\mathop \sum \nolimits_{x} C_{x} /n_{x} }}$$

where *C*_*A*_ corresponds to the number of lipids *A* within a certain cutoff of any HVR (0.55 nm) bead and *n*_*A*_ is the total number of lipids of the specie *A*. Similar to the whole KRAS4b protein, the HVR by itself shows a strong partitioning to anionic lipids (DOPS) (Fig. 6d bottom panel), implying that the co-localization process is in part due to the presence of the polybasic residue stretch (HVR, farnesyl). This demonstrate KRAS4b lateral organization on the membrane is strongly modulated by the tight interaction between the HVR and the anionic lipids, suggesting an important role for the hypervariable region of RAS.

In order to fully understand the forces governing this preferential localization, we attempted to determine the role the HVR and the farnesyl group play in membrane association. First, we calculated the preferential membrane binding of the farnesyl group in either the Lo or Ld regions in terms of potential of mean force (PMF). Biasing both the orientation angle and center of mass (COM) distance between the farnesyl and the membrane allow us to better understand the membrane association mechanism of this chemical group. Although the energy projection maps appear to be independent of the membrane composition, there is a general stabilization of the farnesyl group in the Ld membrane mimic of 5 kJ mol^−1^ (Fig. S4A). Additionally, the farnesyl tumbles more when it is closer to the membrane center, contrary to the more restricted vertical orientation observed in the Lo membrane. This is visualized better when extracting a 1D distance dependent PMF (Fig. S4B). While farnesyl extraction shows a single basin in the Ld case, the presence of both cholesterol and saturated lipids restricts the transition of the farnesyl from the membrane center towards the surface, evidenced by a second basin. Thus, our energetic profile suggests that the tightly lipid-packed environment provided by the Lo regions disfavors the stability of the farnesyl group, which is entropically favored by the less condensed environment in the Ld domain.

Similarly, we also investigated the contribution of the HVR with the anionic lipids in the Ld region. Strikingly, and experimentally validated (Van et al. 2020), the non-farnesylated HVR does not show a strong preference for the membrane, as shown in Fig. S4C. Its membrane association is only enhanced by 2.5 kJ mol^−1^ and comparable to thermal fluctuations. It is interesting that unbiased simulations of the same system report several association-dissociation events within the microsecond time-scale (Fig. S4D). This rapid on and off process supports our energetic calculations and suggests that the highly flexible HVR is required to be physically attached to the farnesyl group to provide a stabilizing mechanism by anionic lipids. In fact, regardless of the G-domain state (occluded vs exposed), the polybasic residues present in the HVR region are able to form stable contacts with the anionic lipids, confirming their role in modulating the attraction towards negatively charge regions of the membrane. Therefore, we conclude that the preference of KRAS4b for the Ld region is mainly attributed to the stabilization of the farnesyl group in such domains, and engagement of the basic residues within the HVR with anionic lipids requires the insertion of the farnesyl group into the bilayer.

## Discussion

Given the prominent role mutant KRAS4b plays in cancer and tumorigenesis, it is important to understand its dynamics on the membrane, as well as the molecular details preceding its interaction with protein effectors. In this work, we have collected several milliseconds of MD trajectories of membrane-bound KRAS4b. The extensive time scale accessible to the CG resolution allows its integration with MSM and HMM analysis for more rigorous analysis of populations and transition rates.

**Fig. 7:**
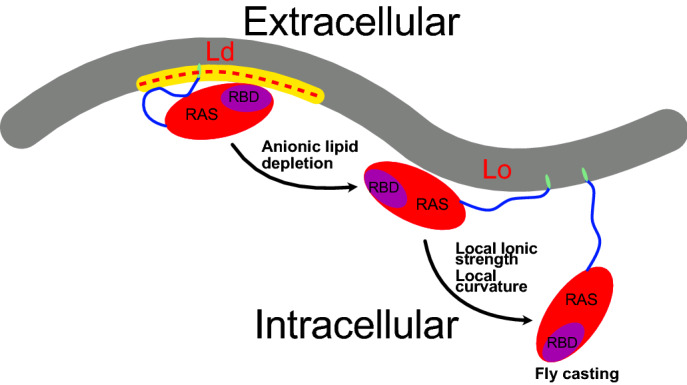
Molecular mechanism of KRAS4b localization on plasma membranes. KRAS4b is highly dynamic and strongly modulated by its interaction with the membrane. High accumulation of anionic lipids in curved Ld domains (Hirama et al. 2017), leads to its localization within these regions. KRAS4b interaction with intracellular effectors (e.g. RAF) is inefficient due to the occlusion of the RAS binding site (RBS). Change in anionic lipid concentration as well as saturated lipids can trigger the transition towards the exposed state. Concerted mechanisms affecting the local ionic strength as well as membrane re-shaping can favor the total detachment of the G-domain (Fly casting)

Our results show KRAS4b is highly dynamic in its membrane-anchored state, alternating fast and slow (ns to μs) between several conformations, and is strongly modulated by membrane lipid composition, the nucleotide-bound state and membrane re-shaping. First, lipid composition appears to be the major determinant for conformational states. Membranes rich in anionic lipids favor the stability of conformations in which the RAF binding site (RBS) is totally hidden (Fig. 7), therefore inaccessible to its effector. This becomes apparent when dynamics in a zwitterionic membrane model are compared to one containing anionic lipids. In zwitterionic membranes, conformations with the total exposure of the RAF binding site is favored, potentially enhancing RAF interaction and subsequent activation of the MAPK cascade. Thus, change in lipid composition is a potential modulatory mechanism for the involvement of RAS during the recruitment of effectors. Interestingly, cancer cells have been observed to modify its total content of anionic lipids (Dobrzynska et al. 2015; He et al. 2015; Hou et al. 2014; Kojima 1993), a behavior that can contribute to an over-activation of the signaling cascade.

The second regulatory mechanism is its activation state; changing the bound nucleotide also impacts its dynamics on the membrane. The GTP-bound KRAS4b favors the occlusion of the RAF binding site, while GDP enhances its exposure. Although this mechanism seems contradictory at first glance, it can prevent the association of KRAS4b with several regulators and effectors (Neale and Garcia 2020). For instance, several experiments have shown that the affinity of RAS by RAF is strongly diminished while in a GDP-loaded state (Chuang et al. 1994; Filchtinski et al. 2010). This mechanism, on the other hand, can potentially stabilize related exchange factors involved in GTP-GDP turnover. Thus, RAS dynamics can have a critical role affecting the regulation of the signaling pathway at several levels.

The third modulatory process is provided by the undulations on the membrane, more precisely, an increase of local curvature due to lipid composition and membrane size. Our results suggest that the population of the membrane-adsorbed occluded state of KRAS4b is decreased by limited interactions with anionic lipids on large membranes. We hypothesize that in larger systems as the ones comparable to biological relevant scales, KRAS4b should exhibit larger dynamics with a decreased stabilization of “membrane-adsorbed” states (e.g. occluded, exposed). Thus, membrane-displaced configurations should be expected to populate in the plasma membrane, increasing the probability of interacting with soluble effectors like RAF (Fig. 7). In fact, a recently published work provides evidences for a steady “membrane-distal” conformation of KRAS4b on large planar membranes (Van et al. 2020). Although such state cannot be directly verified using sized-limited nanoplatforms (e.g. nanodiscs), it is likely that local distorted surfaces will reduce the prevalence of “membrane-closed” conformations, favoring the proposed “fly-casting” mechanism as proposed using Neutron reflectivity experiments (Van et al. 2020).

Not only are our CG simulations are in good agreement with experimental results, they also show good consensus when compared to large scale fully atomistic (AA) simulations already published (Neale and Garcia 2020; Prakash and Gorfe 2019; Prakash et al. 2019). First, at least three different states can be described, with specific regions of the RAS G-domain protein contacting the membrane. In this respect, helices $\alpha$3, $\alpha$4, $\alpha$5 and beta strands $\beta$1, $\beta$2, $\beta$3 play a key role in membrane-protein interactions, overall modulating the access to the bound nucleotide and the RBS. In addition, we provide evidence that HVR and G-domain “intramolecular interactions” are also responsible for modulating these conformations, as proposed before (Prakash et al. 2016). Second, RAS exhibits high dynamics when bound to the membrane. We observe states of rapid transitions in the dynamic landscape space of RAS, consistent with previously published work (Prakash and Gorfe 2019). These regions serve as leading paths between the “exposed” and “occluded” conformations. Surprisingly, transition rates extracted from atomistic simulations suggest at least an order of magnitude difference for the full passage between the major basins (e.g. exposed  ←→ occluded), which are stabilized by ~ 16 kJ mol^−1^, a finding that is in good agreement with our calculations. Given the strong agreement between our CG results and those of AA simulations we propose that using more reductive models, such as CG approaches allows the exploration of different conditions and longer time scales while using less computational resources.

Finally, we observe that KRAS4b preferentially segregates into anionic lipids enriched membrane domains (e.g. Ld), providing an advantage for signaling transduction. Biologically, RAS by itself is not able to translate any extracellular signals, unless it partners with the RAF effector molecule (Hancock 2003; Nussinov et al. 2018). The latter results in dephosphorylation of negative regulatory phospho-sites by the Shoc2-MRAS-PP1 complex followed by trans-phosphorylation of the kinase domain resulting in activation of RAF kinase. Thus, an efficient signaling mechanism would benefit from an enhanced RAS-RAF lateral localization in the membrane. Our past studies suggests that the cysteine-rich domain (CRD) as well as the RBD of RAF are preferentially bound to POPC-POPS (70:30) membranes (Travers et al. 2020, 2018), consistent with experimental observations (Fang et al. 2020; Li et al. 2020; Travers et al. 2018). Thus, if RAF also prefers anionic lipids, it is expected that it will use such lipid enrichment as a potential mechanism for targeting its association with the membrane. In fact, such lateral organization was shown recently, demonstrating the lipid regulatory mechanism on RAS-RAF signaling (Li et al. 2020). Our simulations of KRAS4b in a raft-mimic membrane show that both the farnesyl and the HVR region of KRAS4b favor its preferential localization in Ld regions, where unsaturated anionic lipids are enriched. Thus, evolution has provided an elegant mechanism in which signaling partners do not need to modify their interacting domains to transmit a signal from specific regions of the plasma membrane. Slight modifications in their lipid preference can result in a large variety of cellular environments and a potential mechanism to segregate different signaling pathways.

## Conclusions

In this work, we have used enhanced CG-MSM simulations of KRAS4b in different lipid environments. Our results suggest that membrane composition not only modulates the spatial orientation of RAS but also enhances its co-localization within specific regions of the membrane, potentially leading to RAS recruitment and enrichment. When paired with RAF’s preferential partitioning for anionic regions of the membrane, this mechanism can serve as a signaling amplifier, predominantly modulated by the local re-distribution of the anionic lipid content.

## Supplementary Information

Below is the link to the electronic supplementary material.

Supplementary file1 (PDF 894 kb)

Supplementary file2 (PDF 2738 kb)

Supplementary file3 (PDF 1541 kb)

Supplementary file4 (PDF 908 kb)

## Data Availability

The datasets generated during and/or analyzed during the current study are available from the corresponding author on reasonable request.
