# Supplementary figures and images for: Unveiling the Dynamics of KRAS4b on Lipid Model Membranes
Source: J Membr Biol. 2021 Apr 7;254(2):201–16. doi: 10.1007/s00232-021-00176-z (PMC8052243; doi:10.1007/s00232-021-00176-z)

**A**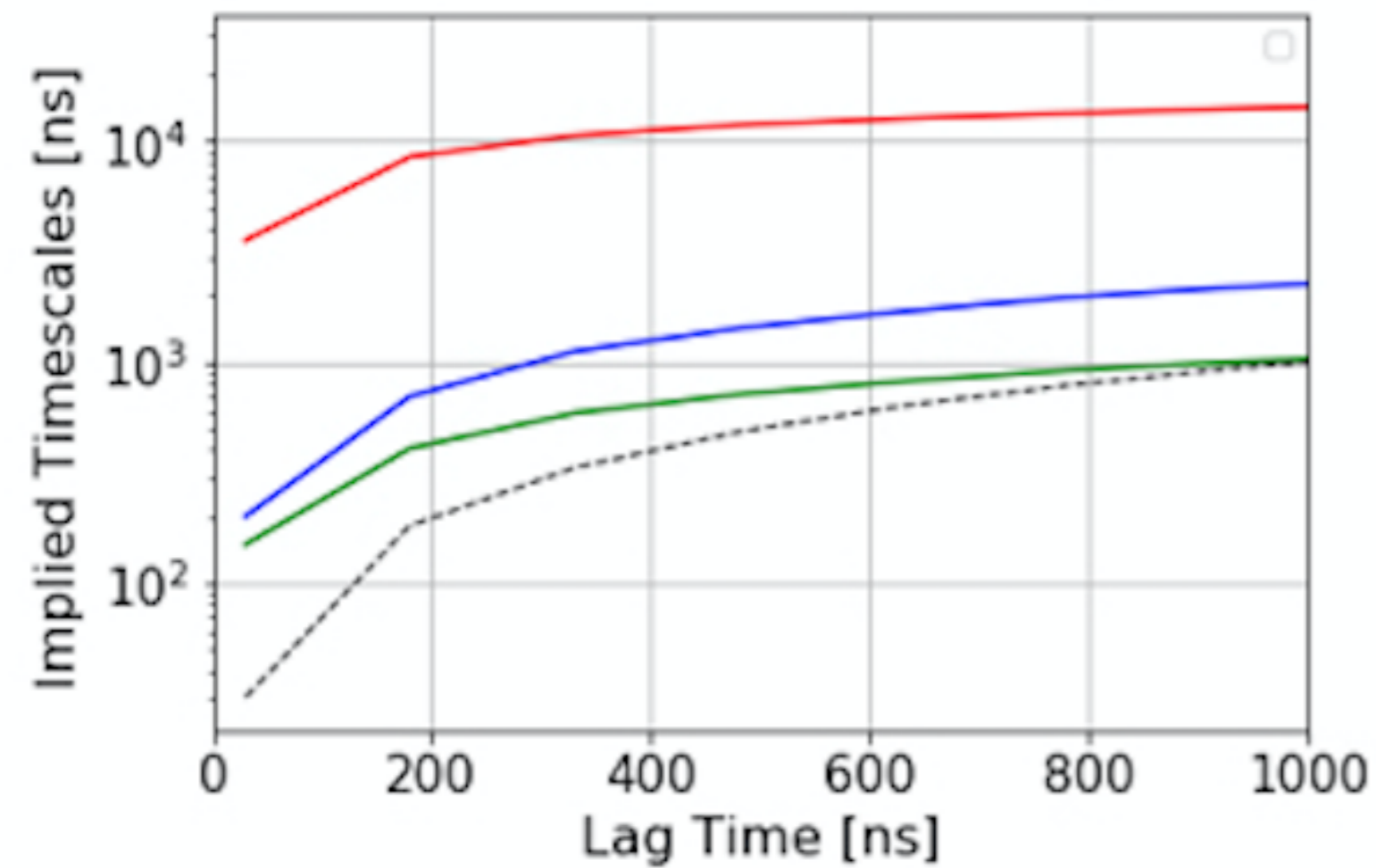**B**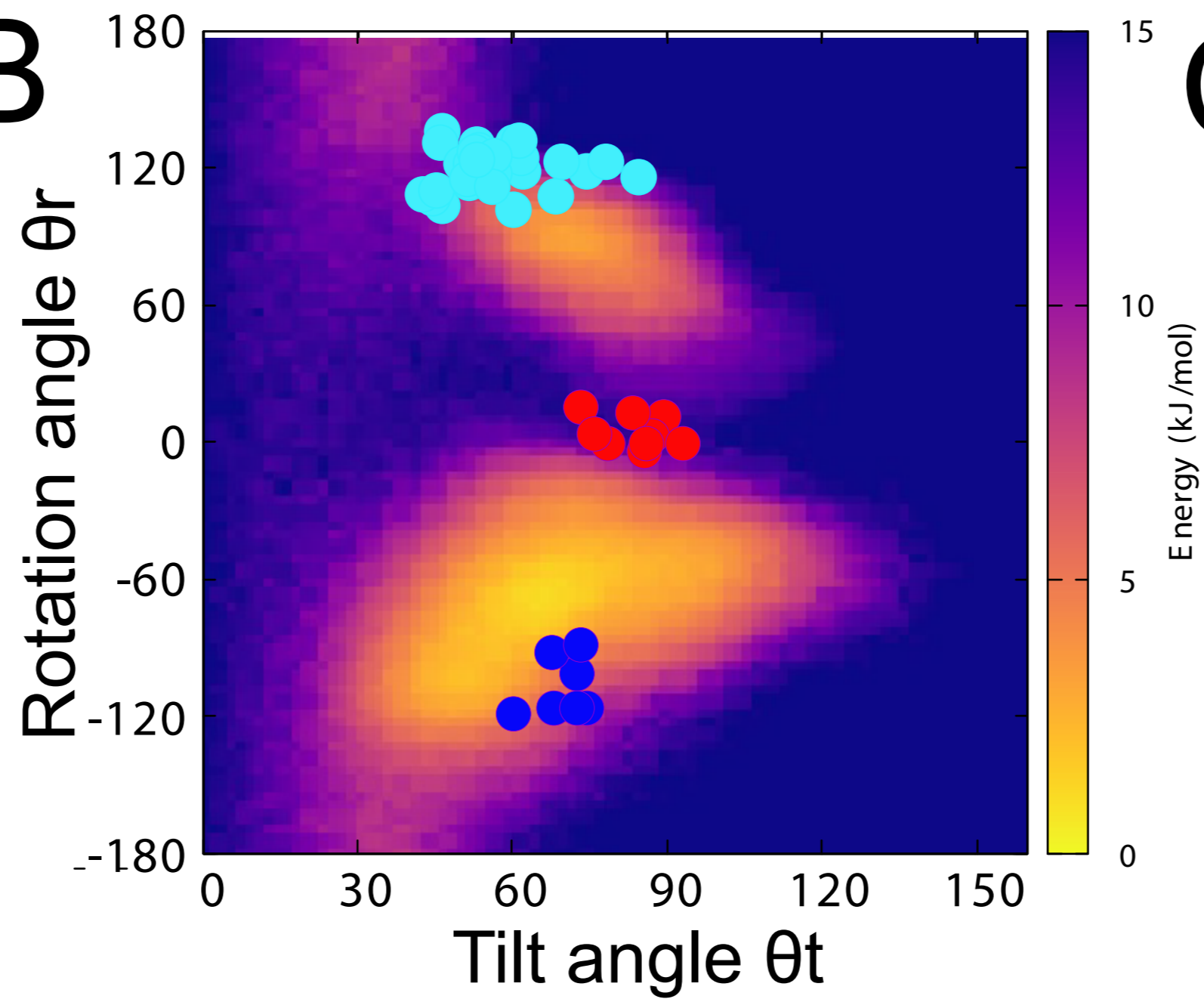**C**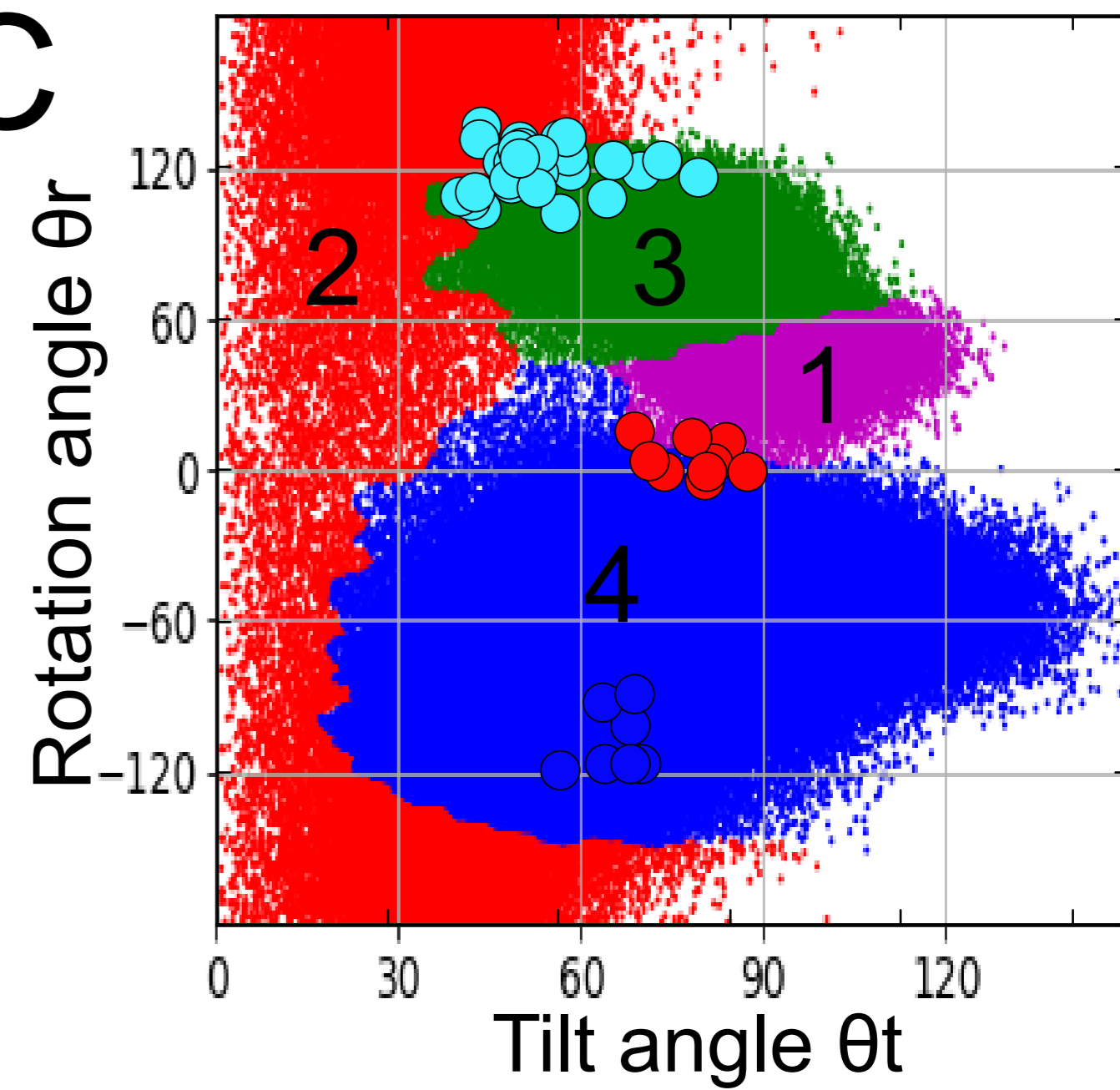

Supplement: Supplementary file 1 — Supplementary file1 (PDF 894 kb) [file 232_2021_176_MOESM1_ESM.pdf]

A

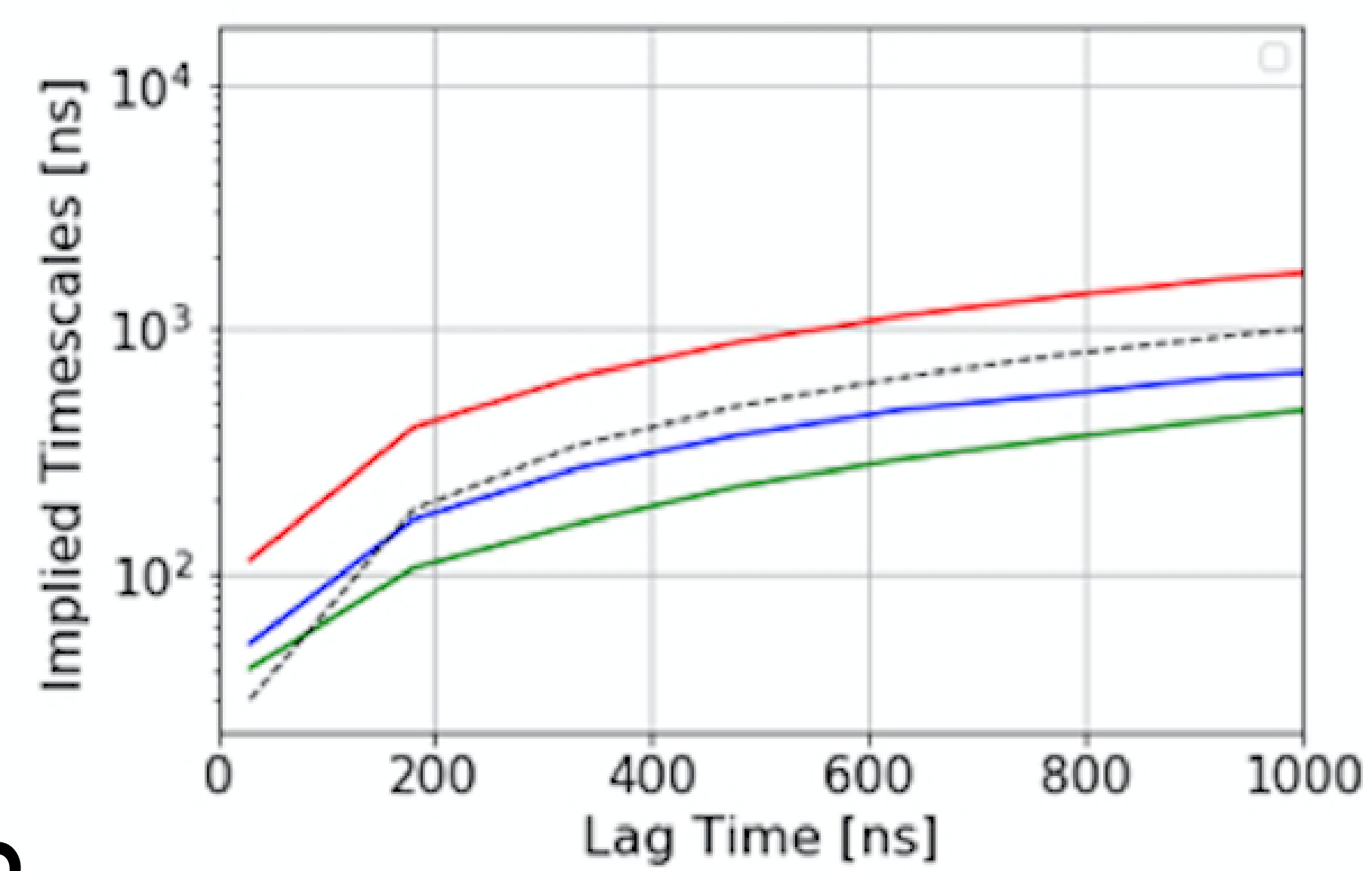

B

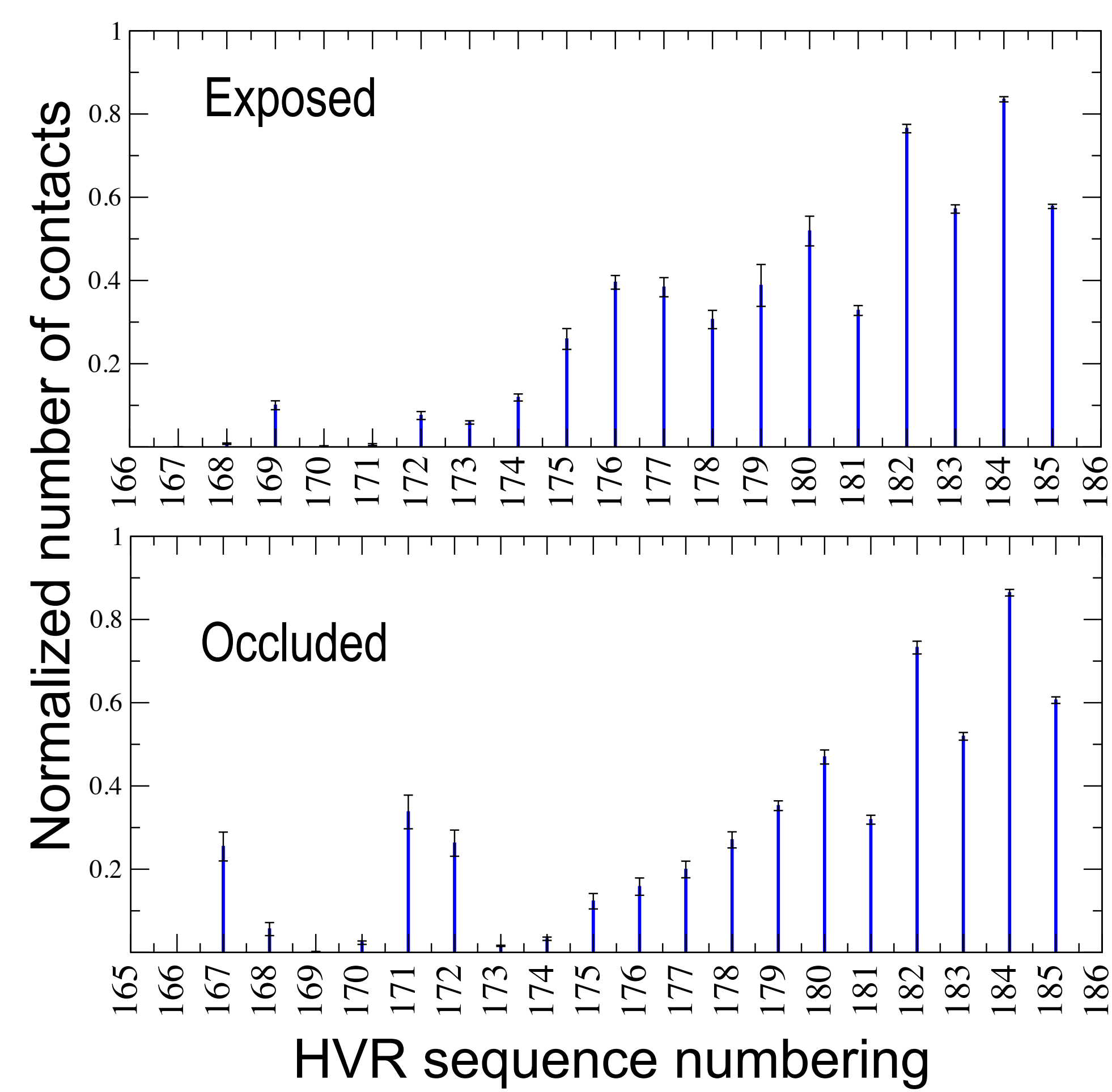

D

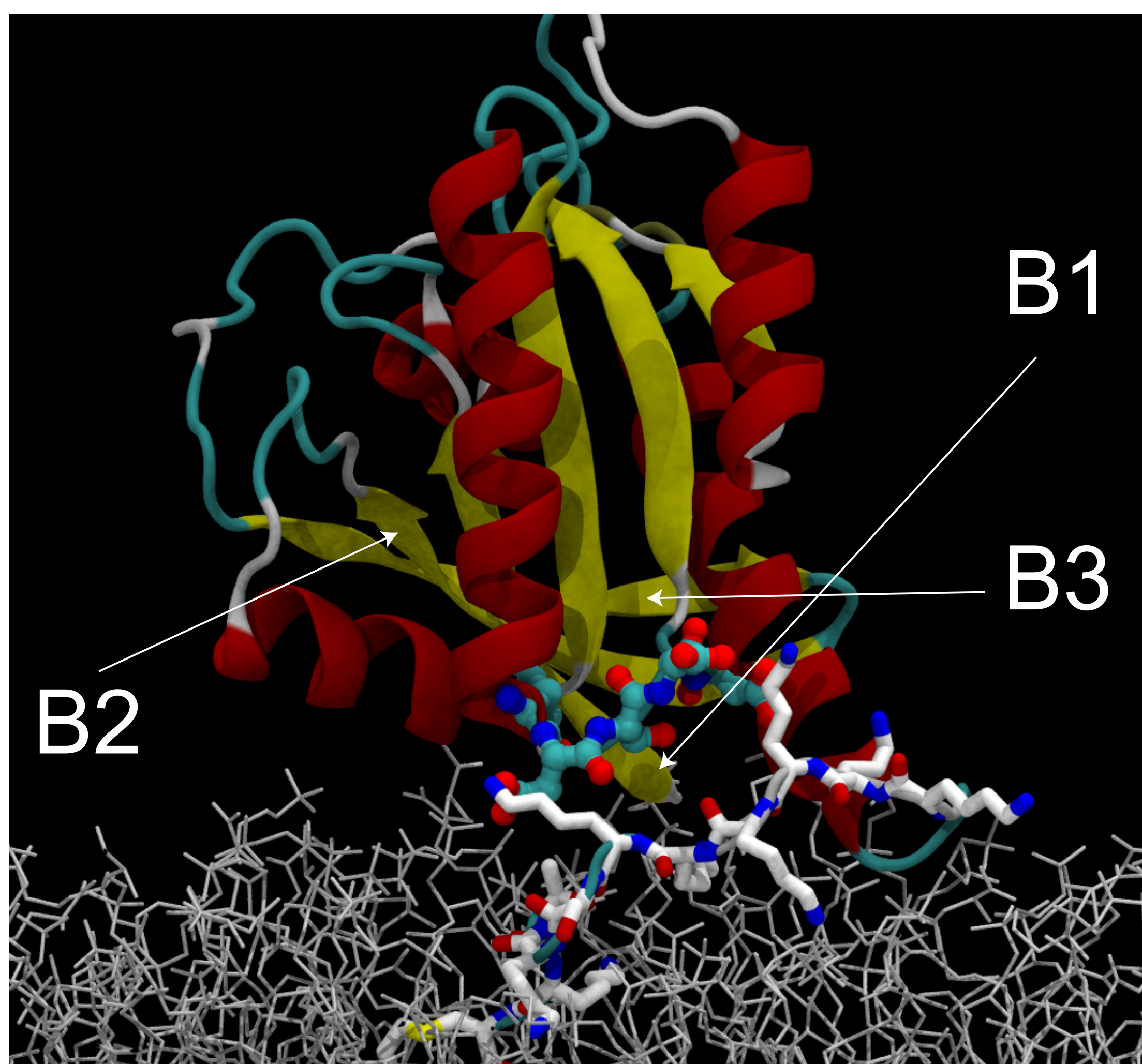

C

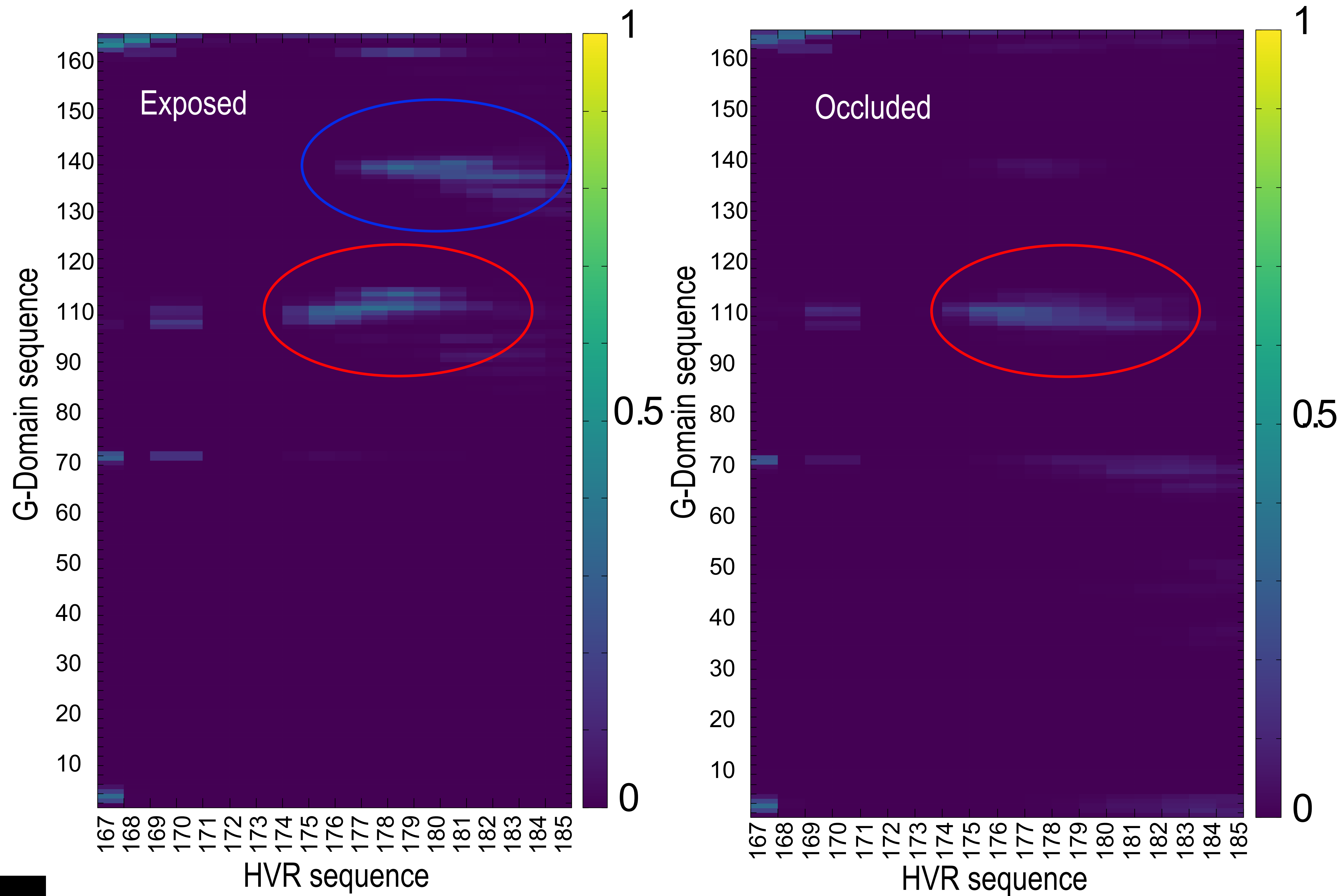

E

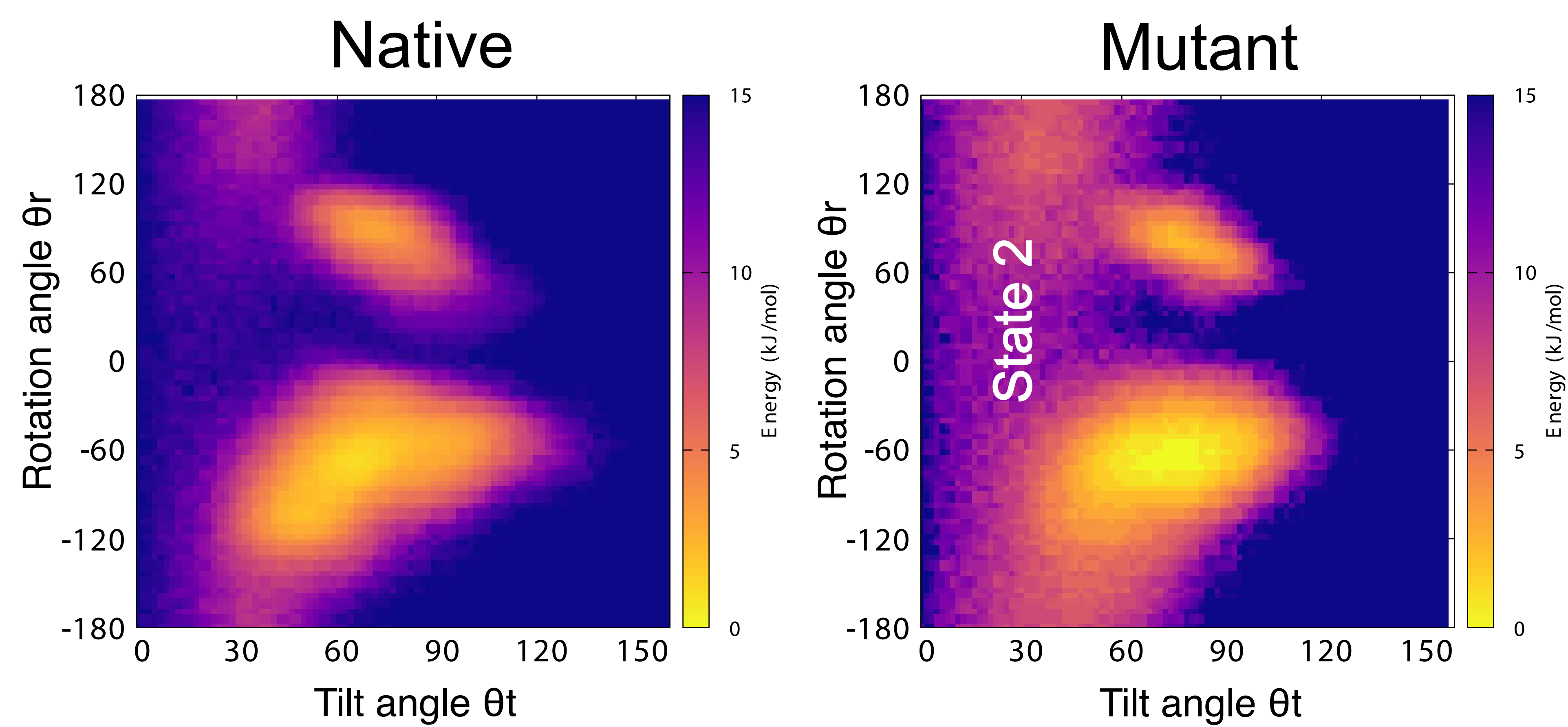

Supplement: Supplementary file 2 — Supplementary file2 (PDF 2738 kb) [file 232_2021_176_MOESM2_ESM.pdf]

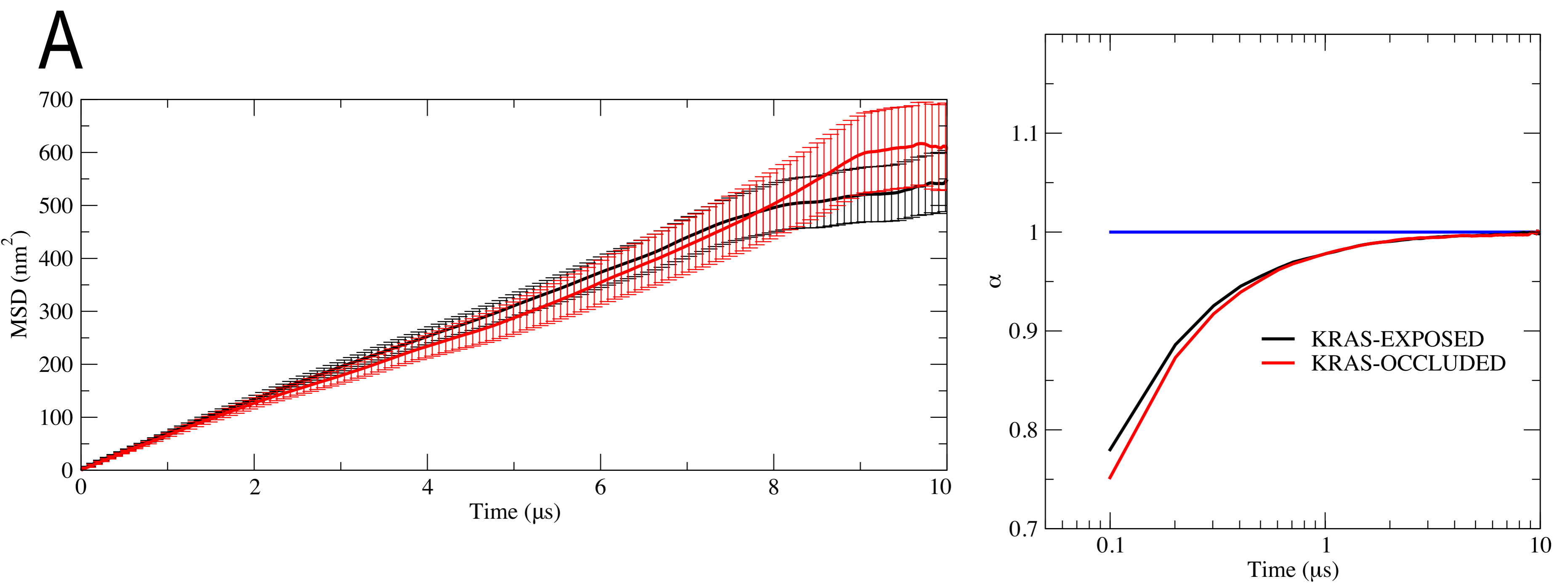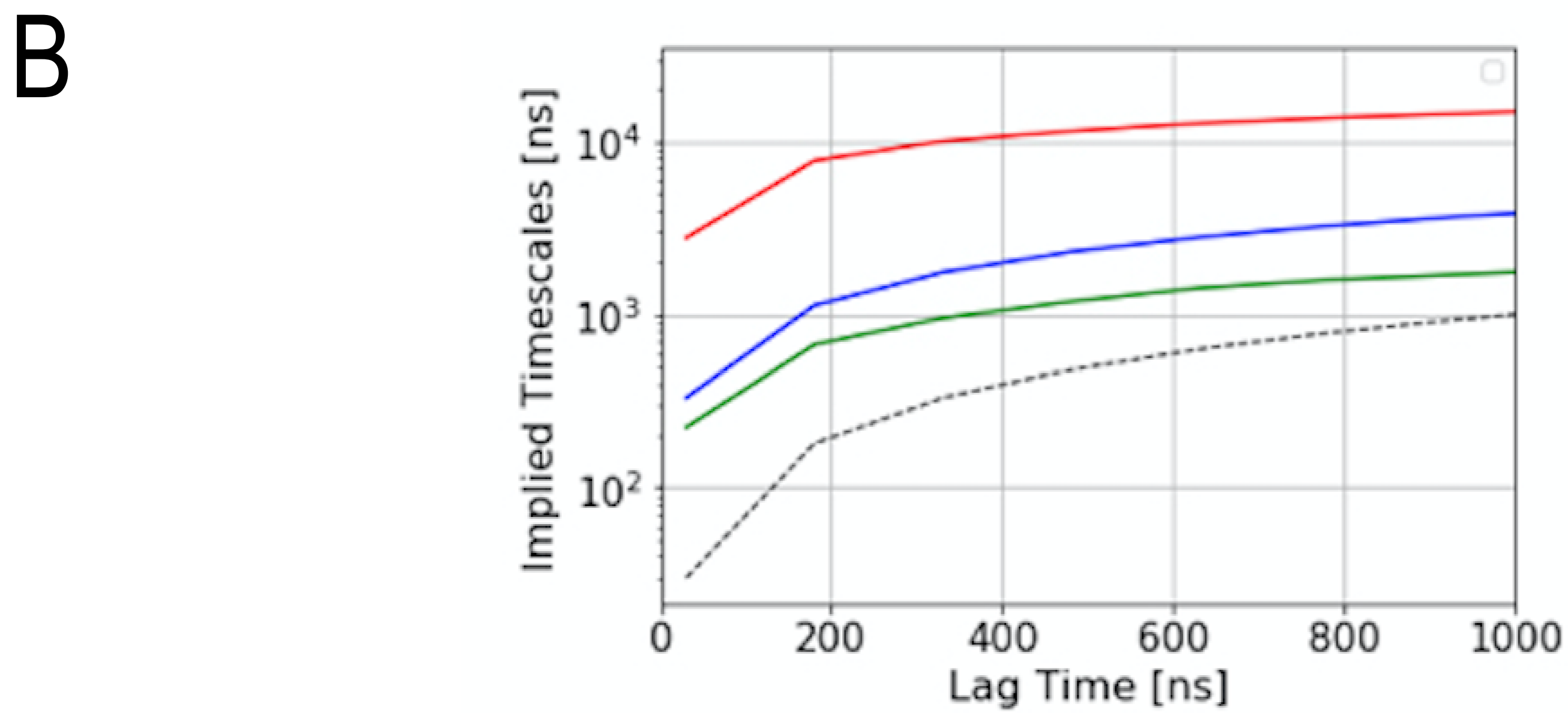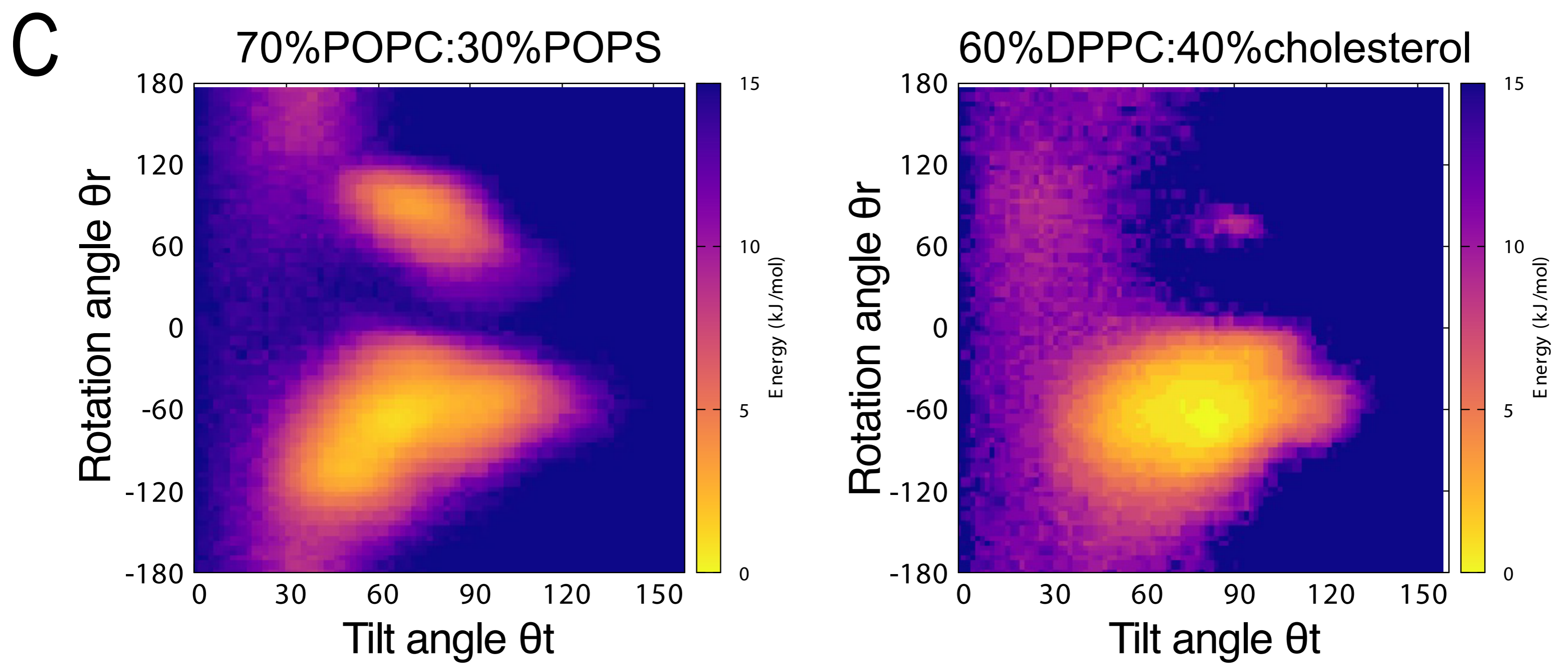

Supplement: Supplementary file 3 — Supplementary file3 (PDF 1541 kb) [file 232_2021_176_MOESM3_ESM.pdf]

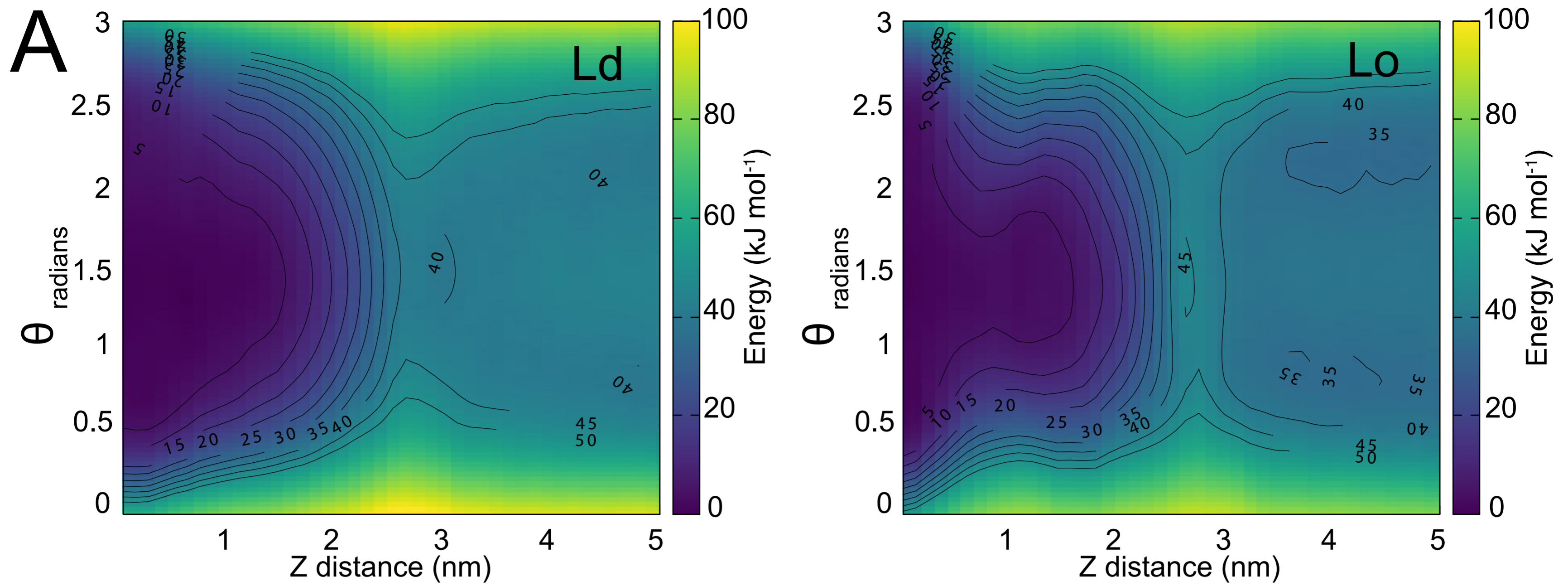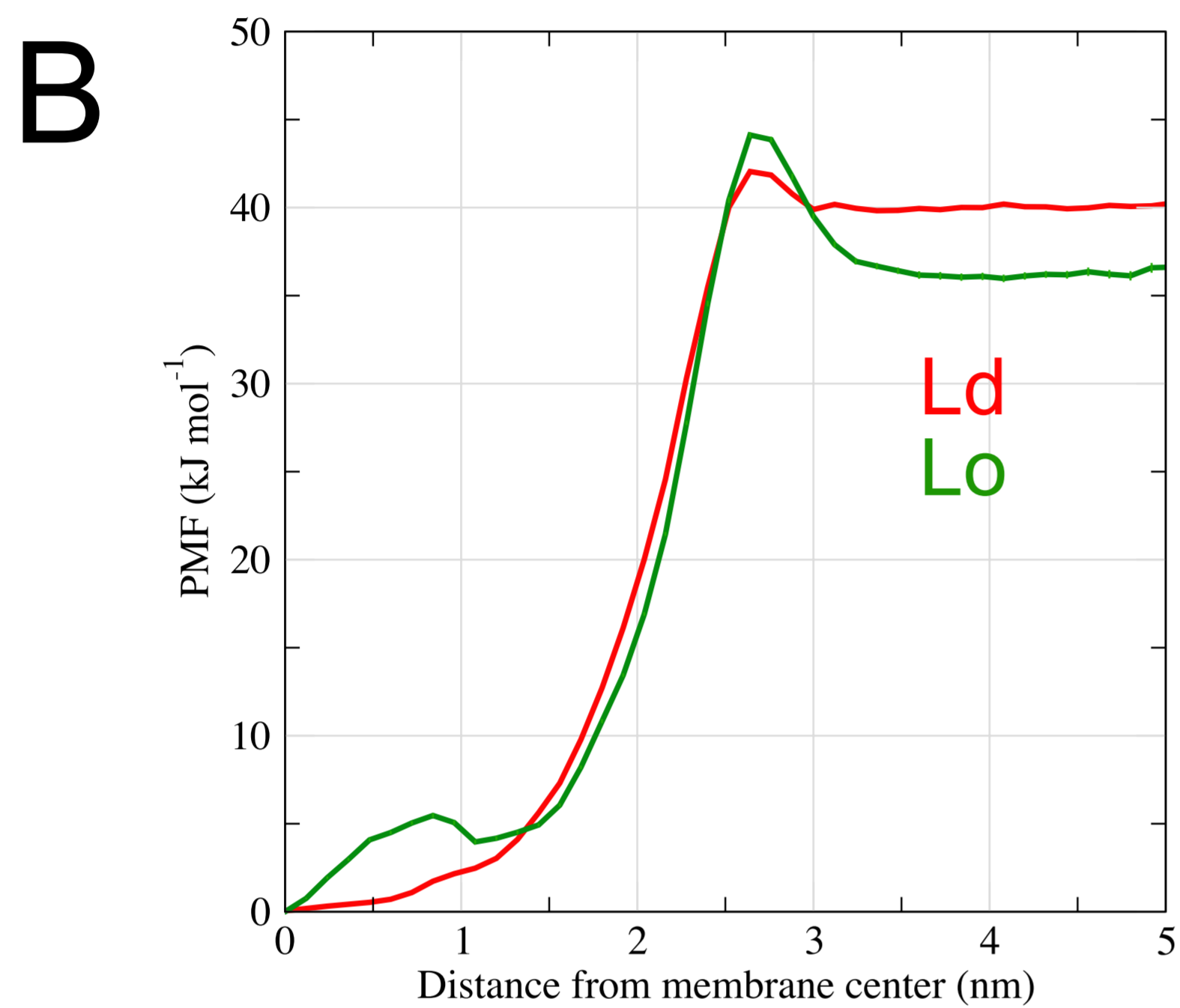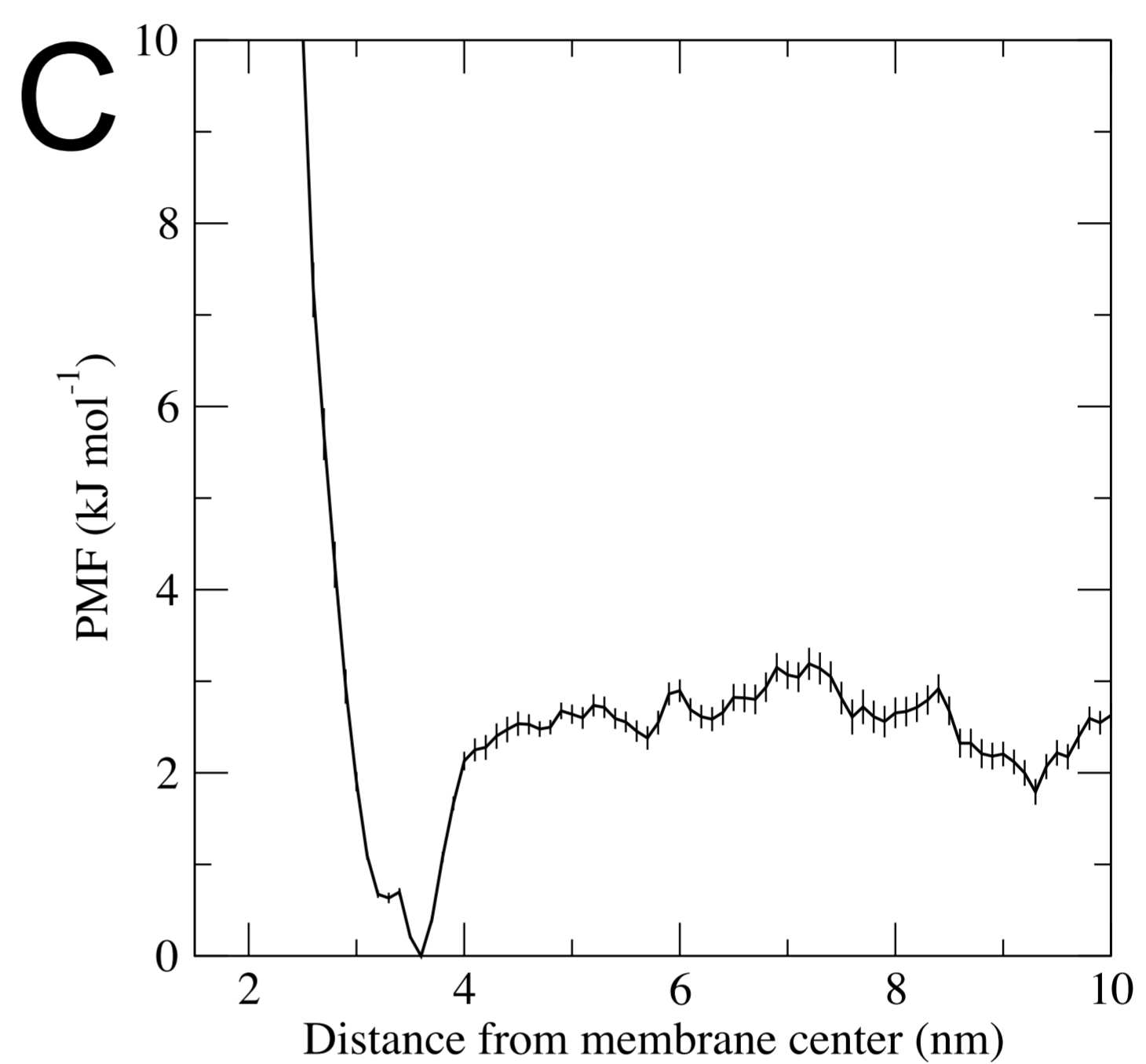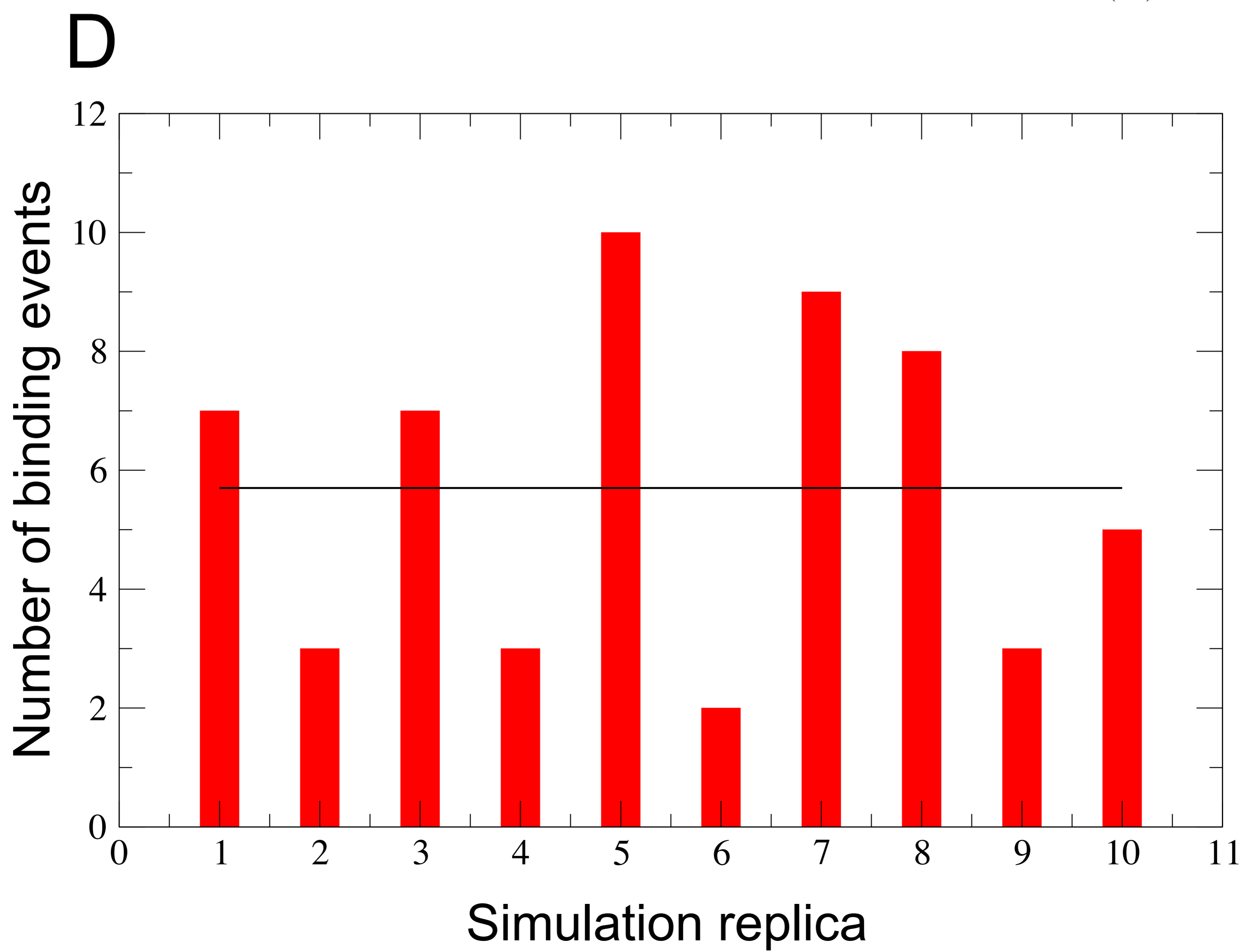

Supplement: Supplementary file 4 — Supplementary file4 (PDF 908 kb) [file 232_2021_176_MOESM4_ESM.pdf]
